# A phase 1/2 trial of an immune-modulatory vaccine against IDO/PD-L1 in combination with nivolumab in metastatic melanoma

**DOI:** 10.1038/s41591-021-01544-x

**Published:** 2021-12-09

**Authors:** Julie Westerlin Kjeldsen, Cathrine Lund Lorentzen, Evelina Martinenaite, Eva Ellebaek, Marco Donia, Rikke Boedker Holmstroem, Tobias Wirenfeldt Klausen, Cecilie Oelvang Madsen, Shamaila Munir Ahmed, Stine Emilie Weis-Banke, Morten Orebo Holmström, Helle Westergren Hendel, Eva Ehrnrooth, Mai-Britt Zocca, Ayako Wakatsuki Pedersen, Mads Hald Andersen, Inge Marie Svane

**Affiliations:** 1 National Center for Cancer Immune Therapy (CCIT-DK), Department of Oncology, Copenhagen University Hospital, Herlev, Denmark; 2 IO Biotech Aps, Copenhagen, Denmark; 3 Department of Clinical Physiology and Nuclear Medicine, Copenhagen University Hospital, Herlev, Denmark; 4 Department of Immunology and Microbiology, University of Copenhagen, Copenhagen, Denmark

**Keywords:** Phase II trials, Melanoma, Translational research, Peptide vaccines

## Abstract

Anti-programmed death (PD)-1 (aPD1) therapy is an effective treatment for metastatic melanoma (MM); however, over 50% of patients progress due to resistance. We tested a first-in-class immune-modulatory vaccine (IO102/IO103) against indoleamine 2,3-dioxygenase (IDO) and PD ligand 1 (PD-L1), targeting immunosuppressive cells and tumor cells expressing IDO and/or PD-L1 (IDO/PD-L1), combined with nivolumab. Thirty aPD1 therapy-naive patients with MM were treated in a phase 1/2 study (https://clinicaltrials.gov/, NCT03047928). The primary endpoint was feasibility and safety; the systemic toxicity profile was comparable to that of nivolumab monotherapy. Secondary endpoints were efficacy and immunogenicity; an objective response rate (ORR) of 80% (confidence interval (CI), 62.7–90.5%) was reached, with 43% (CI, 27.4–60.8%) complete responses. After a median follow-up of 22.9 months, the median progression-free survival (PFS) was 26 months (CI, 15.4–69 months). Median overall survival (OS) was not reached. Vaccine-specific responses assessed in vitro were detected in the blood of >93% of patients during vaccination. Vaccine-reactive T cells comprised CD4^+^ and CD8^+^ T cells with activity against IDO- and PD-L1-expressing cancer and immune cells. T cell influx of peripherally expanded T cells into tumor sites was observed in responding patients, and general enrichment of IDO- and PD-L1-specific clones after treatment was documented. These clinical efficacy and favorable safety data support further validation in a larger randomized trial to confirm the clinical potential of this immunomodulating approach.

## Abstract (web summary)

A first-in-class immune-modulating IDO/PD-L1-targeting vaccine combined with nivolumab in a phase 1/2 trial achieved very promising clinical activity and long-lasting immune responses in patients with advanced melanoma.

## Main

Despite remarkable advances in the treatment of MM with immune checkpoint inhibitors (ICIs) targeting PD-1 and cytotoxic T lymphocyte antigen 4 (CTLA-4), around half of patients are resistant to ICI monotherapy^1^. The combination of anti-CTLA-4 (aCTLA-4) and aPD1 therapy is to date the most effective therapy resulting in a response rate of around 60%; however, 50% of the patients also develop severe adverse events^2,3^. Therefore, an equally effective but less toxic treatment is highly needed. Several approaches to enhance ICI efficacy are currently being investigated, such as other ICIs, T cell therapy with tumor-infiltrating T cells or innate immunity stimulators such as Toll-like receptor 9 agonists^4–6^. Treating cancer patients with vaccines that stimulate a targeted immune response is another attractive approach, with very few side effects observed thus far in combination immunotherapy studies^7,8^.

**Extended Data Fig. 1:**
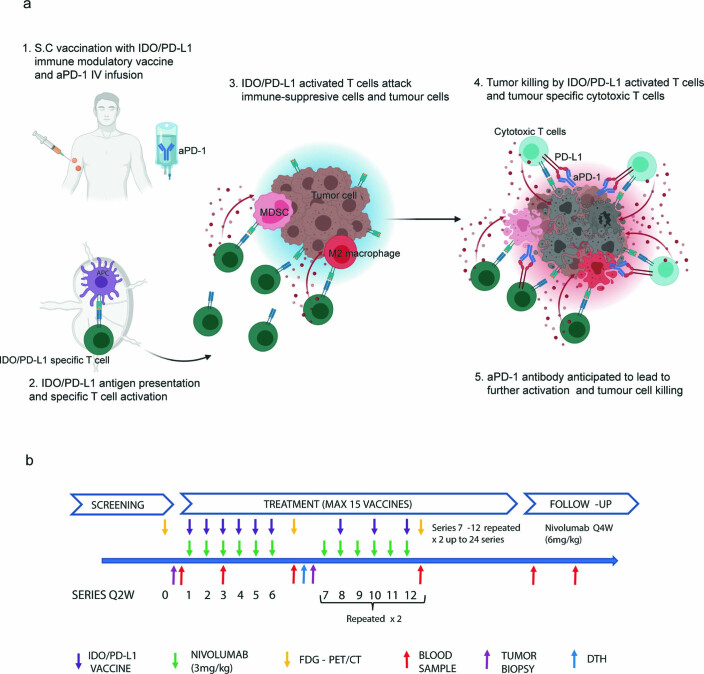
Mode of Action and treatment plan. **a)** Anticipated mechanism of action of the combination therapy of an IDO/PD-L1 derived peptide vaccine and nivolumab (anti-PD1). 1) The IDO/PD-L1 peptide vaccine is administered subcutaneously (s.c) and nivolumab is administered intravenous (IV). 2) The vaccines peptides are phagocytosed by an antigen presenting cell and presented to IDO and PD-L1 specific T cells, which are activated. 3) The activated T cells migrates to the tumor site where they attack both immune-suppressive cells and tumour cells expressing IDO and/or PD-L1 leading to cytokine production and a pro-inflammatory tumour microenvironment. 4/5) Enhanced tumour killing by both IDO/PD-L1 specific T cells and tumor specific cytotoxic T cells due to PD-1 blockade. Created with BioRender.com **b)** Treatment plan. After written informed content patients were screened. Before treatment start a baseline PET/CT scan was performed, baseline blood sample for research use and if assessable a baseline needle biopsy. Patients were treated with the IDO/PD-L1 peptide vaccine subcutaneously biweekly for the first 6 injections and thereafter every fourth week for a maximum of 15 vaccines. Nivolumab was administered in parallel biweekly (3mg/kg) up to 24 series. If patients needed subsequent nivolumab after ended vaccination regiment they were treated with 6 mg/kg every fourth week up to two years. Needle biopsy and delayed type hypersensitivity (DTH) was performed after 6 series of treatment if assessable. PET/CT scans was performed every third month.

Immune-modulatory vaccines targeting tumoral immune escape mechanisms offer a new, generalizable strategy compared to patient-specific neoantigen cancer vaccines^7,9^. The immune-modulatory vaccine strategy in this clinical trial is based on the finding of circulating cytotoxic T cells specific to IDO and PD-L1 in the blood of patients with cancer and, to a lesser extent, in healthy donors. IDO- and PD-L1-specific CD8^+^ T cells can directly recognize and kill IDO^+^ and/or PD-L1^+^ tumor cells and likewise recognize and kill non-malignant cells that express their cognate targets. Furthermore, IDO- and PD-L1-specific CD4^+^ T cells release pro-inflammatory cytokines in response to IDO- or PD-L1-expressing target cells. IDO and PD-L1 are expressed not only by melanoma cells but also by many other cell types in the tumor microenvironment (TME), which differentiates these antigens from traditional tumor antigens used in other studies^10–15^. Activation of IDO/PD-L1-specific T cells by vaccination can therefore restrict the range of immunosuppressive signals mediated by immunosuppressive cells and thereby revert the TME from an immune hostile to an immune friendly environment. In animal models of cancer, vaccinations with IDO epitopes resulted in anti-tumor therapeutic effects that were correlated with reductions in IDO expression in myeloid cell populations within the TME^16^. The IDO/PD-L1 immune-modulating vaccine may lead to a translatable strategy for improving the efficacy of aPD1 therapy through activation of specific T cells. We hypothesize that the IDO/PD-L1 vaccine attracts T cells into the tumor, which induces type 1 helper T (T_H_1) cell inflammation and reverts the TME into an immune-permissive site, thereby turning the tumor ‘hot’. This would also upregulate PD-L1 expression in cancer and immune cells, generating more susceptible targets to aPD1 therapy (Extended Data Fig. 1a). This theory was confirmed in a mouse model in which aPD1 therapy and IDO vaccination show synergistic effects^16^.

In this phase 1/2 clinical trial, MM1636, patients with MM received a combination of the IDO/PD-L1 (IO102/IO103) peptide vaccine with the adjuvant Montanide and the aPD1 antibody nivolumab. Patients were included in three cohorts: 30 aPD1 therapy-naive patients (cohort A), ten aPD1 therapy-refractory patients (cohort B, de novo resistance) and ten patients who progressed after aPD1 therapy (cohort C, acquired resistance). Here, we report results from cohort A.

The vaccine was given biweekly for the first six administrations and thereafter every 4th week. A maximum of 15 vaccines were administered. Nivolumab was given in parallel, biweekly (3 mg per kg) or every 4th week (6 mg per kg) for up to 2 years (Extended Data Fig. 1b).

The primary objective was safety and feasibility. Secondary objectives were immunogenicity and clinical efficacy.

## Results

### Patients and treatment

Thirty patients were enrolled from December 2017 to June 2020. None of the 30 patients dropped out of the study; all received at least three cycles of therapy (Supplementary Fig. 1). At the current database lock (5 October 2020), six patients were still on treatment in the trial.

Of the 24 patients who were not on trial treatment at data cutoff, two are still receiving nivolumab monotherapy (6 mg per kg every 4 weeks). Reasons for stopping treatment for the remaining 22 patients included disease progression (37%), toxicity (20%), maximum benefit or complete response (CR) confirmed on two consecutive scans (17%) or completing 2 years of treatment (7%).

For the 24 patients who were not on trial treatment at data cutoff, the mean number of vaccinations was 10.5 (range, 3–15 vaccinations). Thirteen of these 24 patients continued nivolumab (6 mg per kg, every 4 weeks) as a standard of care. Nine patients received subsequent therapy after progression (Supplementary Table 1).

Baseline characteristics are shown in Table 1. The mean age was 70 years; 37% of patients had elevated lactate dehydrogenase (LDH) levels, 60% were stage M1c, 37% had *BRAF* mutations, and 43% were negative for PD-L1 (<1%). A total of three patients (10%) had received prior ipilimumab therapy. No patients had brain metastasis (Supplementary Table 2).

**Table 1:** Baseline patient characteristics (*n* = 30)

Characteristic	Number (%)
Mean age, years (range)	70 (46–85)
Sex, male	16 (55%)
ECOG PS 0	26 (87%)
LDH levels	
≤ULN	19 (63%)
>ULN	11 (37%)
M stage (AJCC-8)	
M1a	6 (20%)
M1b	6 (20%)
M1c	18 (60%)
Number of lesion sites	
1	6 (20%)
2–3	17 (57%)
>3	7 (23%)
Liver metastases present	10 (33%)
*BRAF* status	
Mutant	11 (37%)
Wild type	19 (63%)
PD-L1	
<1%	13 (43%)
>1%	17 (57%)
Previous systemic therapy	
Ipilimumab	3 (10%)
No	27 (90%)

AJCC-8, eighth edition of the American Joint Committee on Cancer; ECOG PS, Eastern Cooperative Oncology Group performance status; ULN, upper limit of normal.

### Notable clinical responses to the combination therapy

**Fig. 1:**
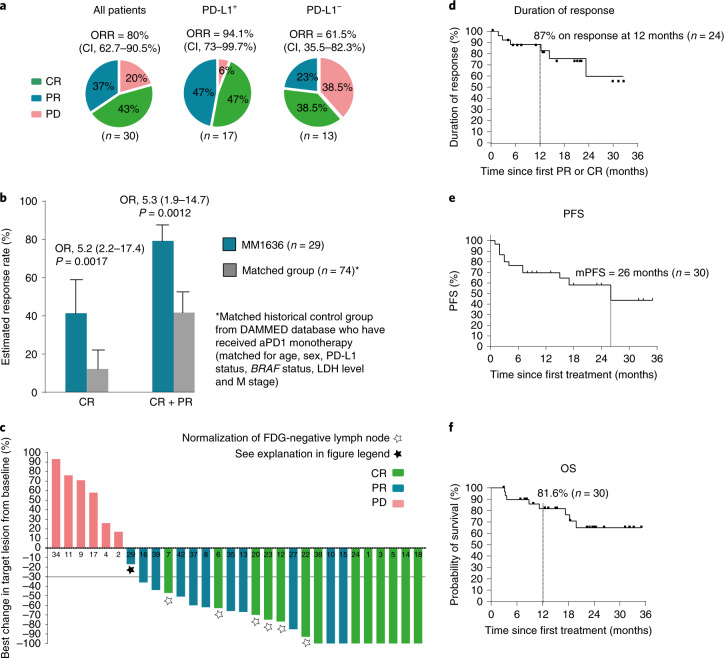
Clinical response. **a**, Pie charts with percent ORR, CR, PR and PD according to RECIST 1.1 by investigator review of all patients (*n* = 30), PD-L1^+^ patients (>1%, (*n* = 17)) and PD-L1^−^ patients (<1%, *n* = 13)), respectively. Two-sided CIs (95%) were constructed using the Clopper–Pearson method. **b**, Treatment effect in MM1636 compared with a matched historical control group from the DAMMED database (*n* = 74). Patients in MM1636 (*n* = 29) were matched with the exact same combination variable according to age (≤70 years, >70 years), sex, LDH levels (normal, elevated), M stage (M1a, M1b, M1c), *BRAF* status (wild type, mutated) and PD-L1 status (<1%, ≥1%). Estimates for treatment effects were calculated by weighted logistic regression analyses and weighted Cox proportional hazard model. Bar height indicates the estimated response rate; tops of bars are centers for error bars. Odds ratios (OR), response rates and their corresponding 95% CIs were extracted from the regression model. All *P* values were two sided, and *P* values below 0.05 were considered statistically significant. **c**, Best change in the sum of target lesion size compared with that at baseline (*n* = 30). The horizontal line at −30 shows the threshold for defining an objective response in the absence of non-target disease progression or new lesions according to RECIST 1.1. Two patients with 100% reduction in target lesion size had non-target lesions present. White stars, six patients had normalization (<10 mm) of fluor-18-deoxyglucose (FDG)-negative lymph nodes (at baseline, lymph nodes were >1.5 cm and FDG^+^) and 100% reduction of non-lymph node lesions and are considered to have had a CR (green bar). Black star, one patient (MM29) was considered to have had a PR (blue bar), although he had not reached a −30% change in target lesion size. The patient had a single measurable 13-mm lung metastasis at baseline and multiple biopsy-verified cutaneous metastases on the left crus (not detectable by positron-emission tomography–computed tomography (PET–CT) at baseline). The best change in target lesion size was 10 mm, and a post-treatment biopsy from the cutaneous metastases showed no sign of malignancy. Thus, overall, the patient was classified as having a PR. **d**, Kaplan–Meier curve of the response duration in the 24 patients with an objective response. **e**, Kaplan–Meier curve of PFS in all 30 treated patients. **f**, Kaplan–Meier curve of OS in all 30 treated patients.

**Fig. 2:**
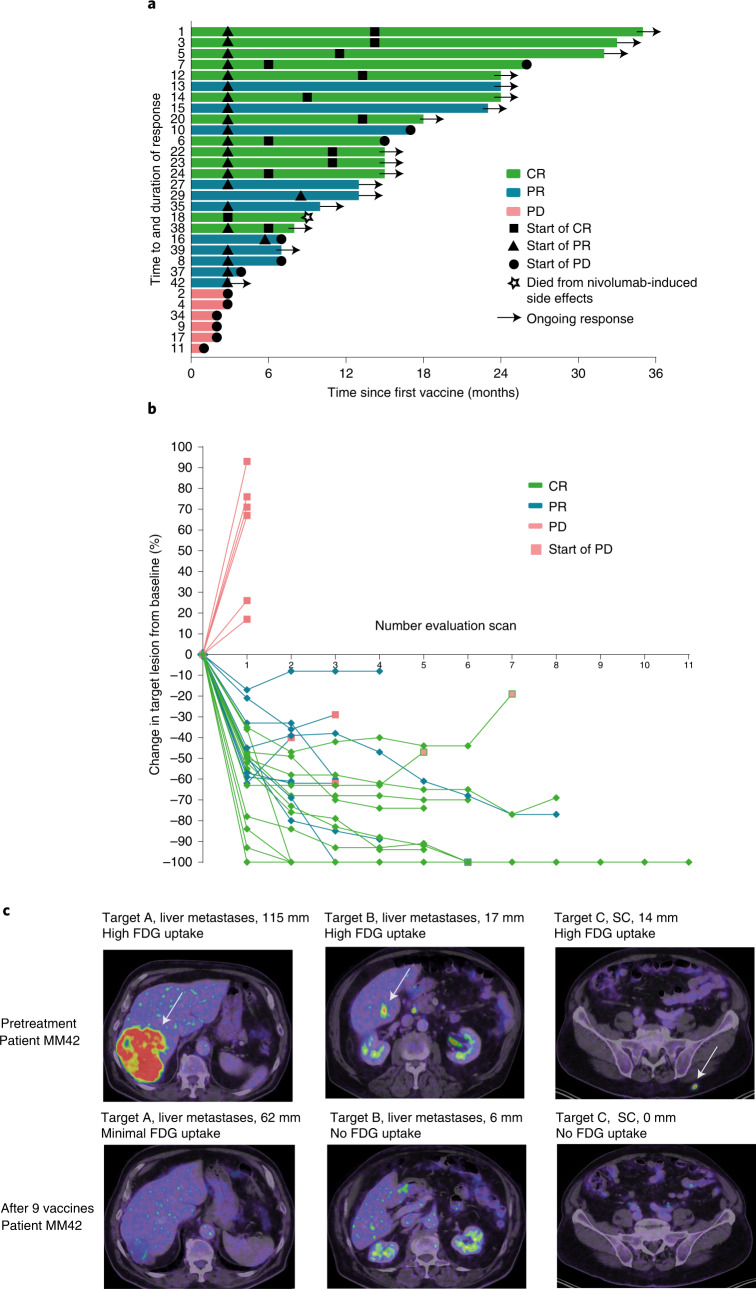
Clinical response. **a**, Swimmer plot showing response duration and time to response according to RECIST 1.1 for all treated patients (*n* = 30). Triangles indicate first evidence of PR, while squares indicate first evidence of CR. Closed circles indicate time of progression. Arrows indicate ongoing responses. Patient MM18 died due to nivolumab-induced side effects. **b**, Spider plot showing response kinetics in all treated patients (*n* = 30). Red squares indicate time of progression. **c**, PET–CT images of patient MM42 before and after treatment (after 12 series of treatment) showing FDG metabolism in target lesions. SC, subcutaneous.

Thirty patients with MM were treated with the IDO/PD-L1 vaccine and nivolumab according to the trial protocol. By investigator review, the ORR reached 80% (CI, 62.7–90.5%), with 43% of patients (CI, 27.4–60.8%) achieving a CR and 37% (CI, 20.9–54.5%) reaching a partial response (PR) as the best overall response, while 20% experienced progressive disease (PD) according to Response Evaluation Criteria in Solid Tumors (RECIST) 1.1 (Fig. 1a). Two of the patients with a PR did not have a confirmatory scan stating PR on two consecutive scans. Early onset of response was frequent, with 22 of 30 patients having an objective response at the first evaluation (after 12 weeks on treatment). Median times to PR and CR were 75 d (range, 54–256 d) and 327 d (range, 73–490 d), respectively (Fig. 2a–c).

The ORR among PD-L1^+^ (>1% (clone 28.8)) patients (*n* = 17) was 94.1% (CI, 73–99.7%) and 61.5% (CI, 35.5–82.3%) in PD-L1^−^ patients (*n* = 13) (Fig. 1a). Objective responses were observed in patients irrespective of human leukocyte antigen (HLA) genotype (Supplementary Fig. 2).

Clinical response data were validated by blinded independent external review, in which an ORR of 76.6% (CI, 57.7–90.1%) was reported, with 53.3% of patients achieving a CR, 23.3% achieving a PR and 3.3% experiencing stable disease. Comparisons between investigator review and external review are outlined in Supplementary Table 3.

To examine whether the very high response rate should be attributed to nivolumab or to the vaccine, we retrieved clinical response information from a matched historical control group using the Danish Metastatic Melanoma Database (DAMMED) from contemporaneously treated patients with stage 3–4 melanoma who received aPD1 monotherapy^17^. Patients were matched with the exact same combination variable according to age, sex, PD-L1 status, *BRAF* status, LDH level and M stage (those at stage M1d were excluded from the control group (no patients with brain metastasis)). Matched controls were identified for 29 patients, and the ORR of 79.3% (CI, 61.0–90.4%) observed in MM1636 was found to be significantly higher (*P* < 0.0012) than that in the matched control group in which an ORR of 41.7% (CI, 31.0–53.3%) was reached. Furthermore, of the 29 patients in MM1636, a significantly (*P* < 0.0017) higher percentage (41.4% (CI, 25.2–59.6%)) of patients achieved CR in MM1636 than that (12% (CI, 6.3–21.6%)) in the matched historical control group. ORR and complete response rate (CRR) in the matched historical control group were comparable to those of patients treated in randomized phase 3 pivotal trials with aPD1 monotherapy^18^ (Fig. 1b).

### The treatment leads to prolonged PFS

At data cutoff, the median duration of response had not been reached, with 87% of all responding patients being free from progression at 12 months (Fig. 1d).

Patients were followed for up to 35 months with a median follow-up time of 22.9 months (CI, 14.9–26.2 months). OS and PFS were calculated from the first day of treatment to death or progression or to the date of the last follow-up (5 October 2020).

**Extended Data Fig. 2:**
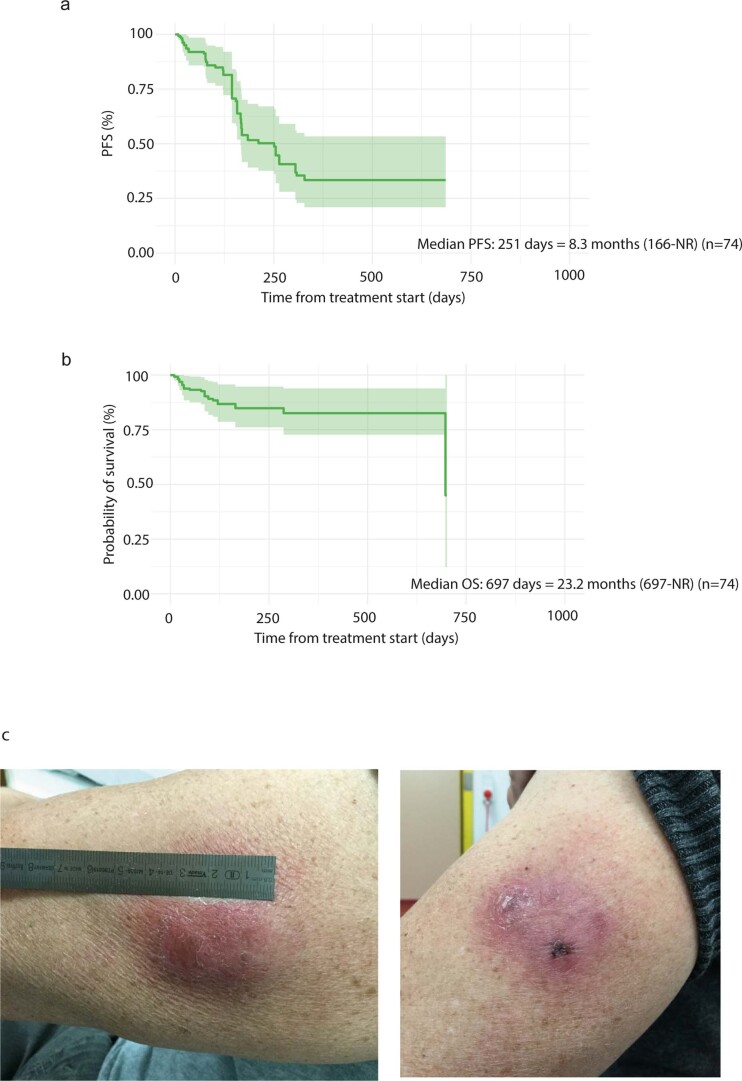
Progression free survival and overall survival in matched historical control group and vaccine injection site reaction. **a**) Kaplan-Meier curve of progression free survival with corresponding 95% confidence intervals in the matched historical control group (n=74). Patients were matched on BRAF-status, PD-L1-status, age, gender, M-stage and LDH level). **b)** Kaplan-Meier curve of overall survival with corresponding 95% confidence intervals in the matched historical control group (n=74). **c)** Images of injection site reaction in patient MM20 (CR) after 11 vaccination show redness, rash and granuloma at injection site.

The median PFS (mPFS) for all treated patients was 26 months (CI, 15.4–69 months) and was not reached for responding patients (Fig. 1e). The median OS was not reached at the data cutoff. OS at 12 months was 81.6% (CI, 61.6–92%) (Fig. 1f). One patient (MM18) with a CR died from nivolumab-related severe adverse events; the remaining patients died because of metastatic melanoma. For comparison, the mPFS was 8.3 months (CI, 5.5 months–NR (not reached)) in the matched historical control group (*n* = 74), while the median OS was 23.2 months (CI, 23.2 months–NR). (Extended Data Fig. 2a,b).

### The combination of the IDO/PD-L1 vaccine and nivolumab was safe

Treatment-related adverse events are listed for all 30 patients in Supplementary Table 4. Common treatment-related grade 1–2 toxicities were fatigue (47%), rash (47%), arthralgia (30%), diarrhea (23%), nausea (23%), dry skin (20%), pruritus (20%), infusion reaction (17%), xerostomia (17%) and myalgia (17%).

Four patients (13%) experienced grade 3–4 adverse events: one patient with a grade 3 maculopapular rash (MM01), one patient with grade 3 adrenal insufficiency (MM06) and one patient with grade 3 arthralgia (MM22).

Patient MM18 died from urosepsis with multi-organ failure and severe hyponatremia. This patient had experienced multiple immune-related adverse events with grade 3 colitis, grade 2 pneumonitis, grade 3 arthralgia, grade 2 vasculitis and grade 2 nivolumab infusion-related allergic reaction. Additionally, patient MM18 had symptoms of myocarditis at the time of death with highly elevated cardiac troponin I levels. Bedside echocardiography showed an ejection fraction of 15%, which at baseline was 60%, but an autopsy was not conducted, and myocarditis was never confirmed pathologically.

Patient MM06 had received first-line treatment with ipilimumab before entering the trial and was on substitution corticosteroids at the time of inclusion. Adrenal insufficiency was aggravated by an erysipelas infection with high fever, reaching grade 3 in Common Terminology Criteria for Adverse Events and resolving quickly after appropriate antibiotic therapy was initiated.

As expected, local side effects were common with 77% of the patients who developed injection site reactions. These reactions were classified as granulomas (63%), redness (20%), pain (13%) and pruritus (13%) at the injection site. All local reactions were grade 1–2, most likely related to the Montanide adjuvant and typically transient. However, two patients (MM07 and MM20) decided to discontinue vaccination after eight and 11 injections, respectively, due to granulomas, tenderness and pain that limited instrumental activities of daily living but continued to receive nivolumab (Extended Data Fig. 2c).

### Vaccine-specific responses in blood were frequently detected

First, all 30 patients were assessed for the presence of vaccine-specific responses in peripheral blood mononuclear cells (PBMCs) before, on and after vaccination using a modified interferon (IFN)-γ enzyme-linked immune absorbent spot (ELISPOT) assay. This assay is known to expand antigen-specific memory cells and to improve the detection and the correlative power of ELISPOT during treatment^19–21^.

**Fig. 3:**
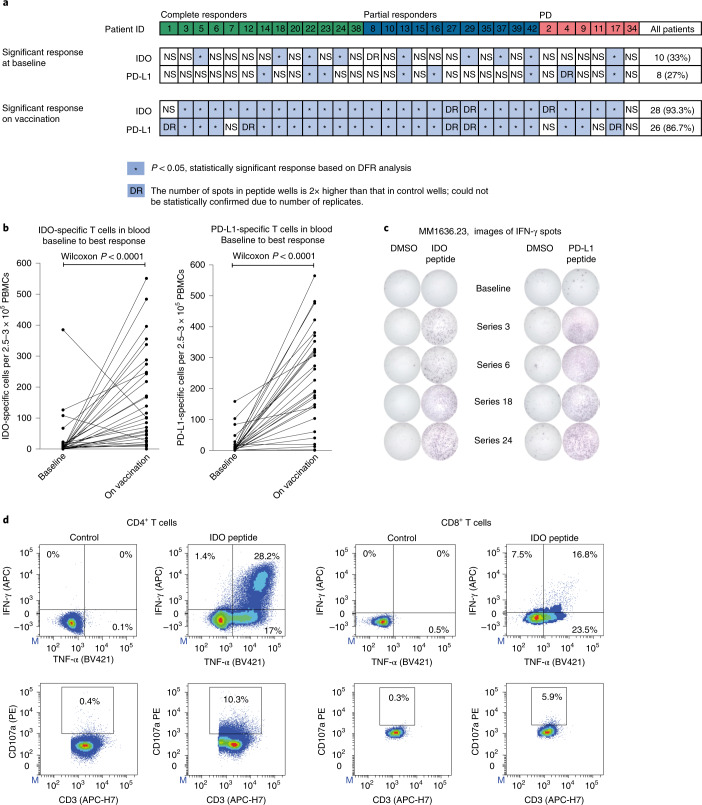
Vaccine-specific responses in blood. **a**, IDO- and PD-L1-specific T cell responses in PBMCs at baseline and on vaccination as measured by the IFN-γ ELISPOT assay (*n* = 30). *Responses were calculated as the difference between the average numbers of spots in wells stimulated with IDO or PD-L1 peptide (triplicates) and those from the corresponding control (DMSO), and statistical analyses of ELISPOT responses were performed using a distribution-free resampling method (Moodie et al.^50^). DR (double response), response was not statistically confirmed due to replicate number, but the number of spots in peptide wells was two times higher than that in control wells (DMSO). NS, no significant response and no DR. For a detailed overview of responses at serial time points on vaccination, see Extended Data Fig. 4a. **b**, IDO- and PD-L1-specific T cell response in PBMCs in all treated patients measured by the IFN-γ ELISPOT assay at baseline and on vaccination. On-vaccination responses were selected from the ‘best’ ELISPOT response at different time points for each patient (series 3, 6, 12, 18 or 24) during vaccination (*n* = 30). Wilcoxon matched-pairs signed-rank test was used to compare responses to IDO or PD-L1 peptides in the vaccine between baseline and later time points. **c**, Representative example of ELISPOT wells with response in patient MM23 in serial PBMCs before and on treatment. **d**, IDO-specific CD4^+^ and CD8^+^ T cells were isolated and expanded from PBMCs stimulated in vitro with the IDO peptide and a low dose of IL-2 for 14–15 d before sorting using the Miltenyi Cytokine Secretion Assay—Cell Enrichment and Detection kit. To assess their cytolytic potential, IDO-specific T cells were stimulated with IDO peptide, and expression of CD107a, IFN-γ and TNF-α was assessed by flow cytometry (the example is from patient MM14).

**Extended Data Fig. 3:**
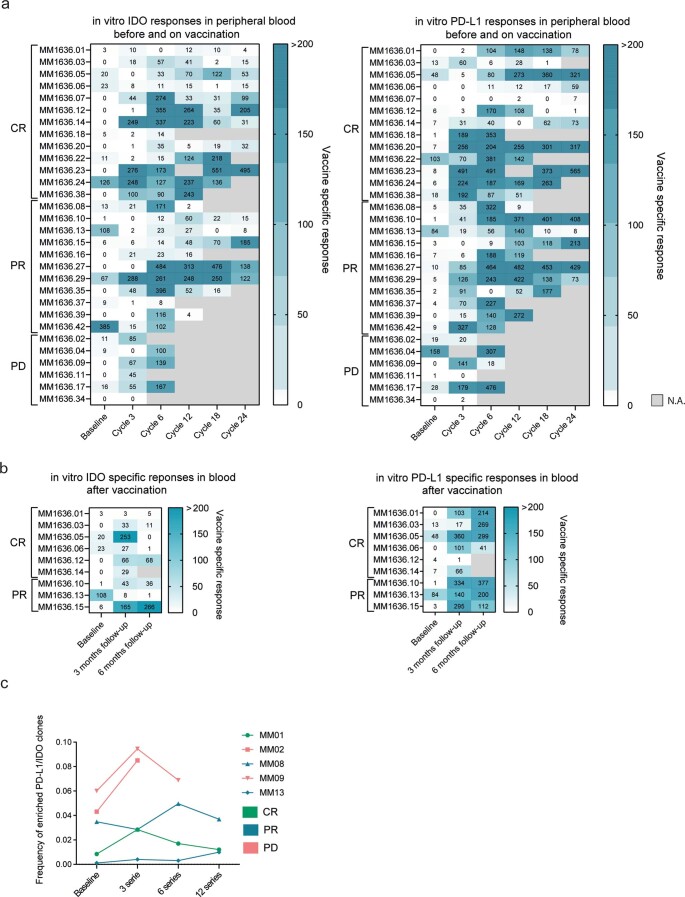
Vaccine specific responses in blood. **a)** Heatmap of specific (background has been subtracted) IDO and PD-L1 responses in PBMCs at baseline, series 3, 6, 12, 18 and 24 measured by in vitro IFN-γ Elispot assay show fluctuations in the blood during treatment (n=30). **b)** Heatmap of specific (background has been subtracted) IDO and PD-L1 responses in PBMCs at baseline and 3 and 6 months after last vaccine measured by in vitro IFN- γ Elispot assay (n=30). 2.5–3.2x10^5^ cells per well were used. **c)** Vaccine associated clones were tracked in the blood by summing the frequency of each rearrangement enriched in either IDO or PD-L1 T cells.

**Extended Data Fig. 4:**
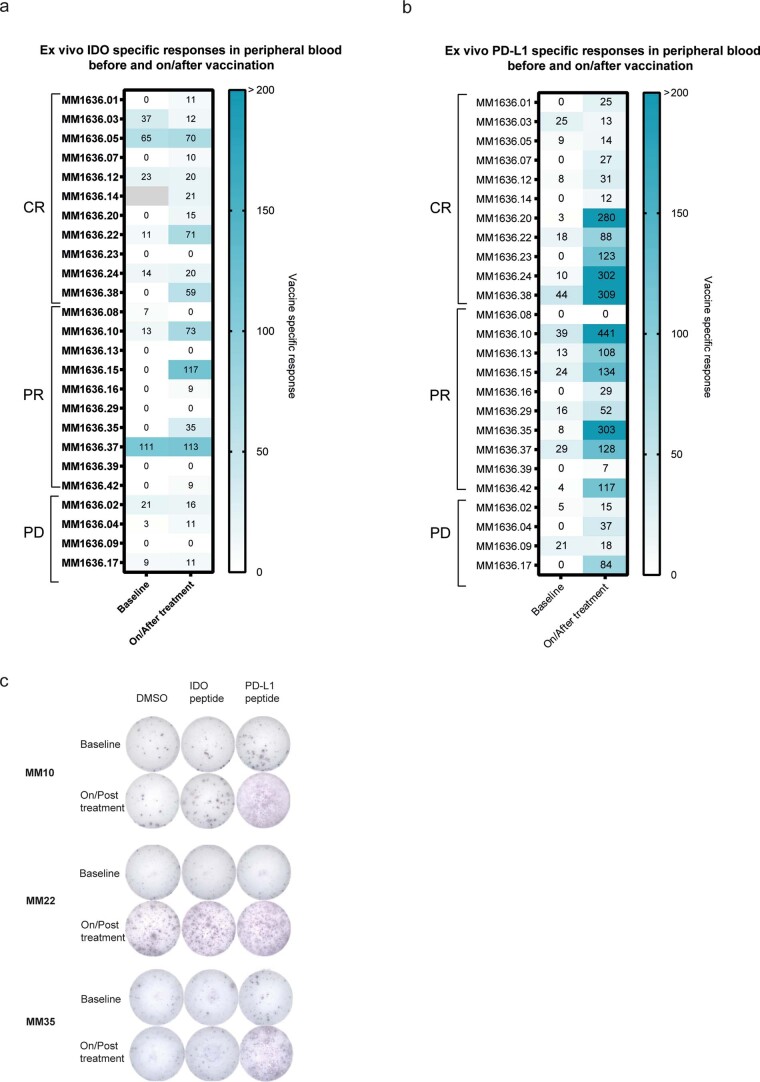
Ex vivo vaccine specific responses in blood. Heatmaps of detected specific (background has been subtracted) IDO (a) and PD-L1 (b) responses in PBMCs at baseline and on/after treatment as measured by IFNγ ELISPOT (n=25). C) Example of well images of ex vivo ELISPOT wells for three different patients (n=3). 6-9x10^5^ cells per well were used.

Prevaccine IDO-specific responses were detectable in ten (33%) patients, while prevaccine PD-L1-specific responses were detectable in eight patients (27%); overlapping (specific to both IDO and PD-L1) prevaccine responses were present in four (13.3%) patients. During vaccination, an increase of IDO-specific T cells or PD-L1-specific T cells in the blood was observed in 28 (93%) and 26 (86%) patients, respectively. In total, 93% of patients had an increase in either PD-L1- or IDO-specific responses on vaccination (Fig. 3a), with a significant (*P* < 0.0001) median increase from baseline to the post-vaccine response demonstrated for both IDO and PD-L1 (at different time points on treatment), confirming that vaccine-specific immune responses were induced in patients regardless of clinical response (Fig. 3b,c). Immune responses fluctuated in the blood over time (Extended Data Fig. 3a). An increase in IDO- and PD-L1-specific responses in the peripheral blood was also detectable directly ex vivo across the different clinical response groups (Extended Data Fig. 4).

Sustained vaccine-specific responses were observed 3 and 6 months after the last vaccine, indicating induction of memory responses in nine patients with response to clinical treatment who surpassed follow-up at data lock (Extended Data Fig. 3b). Importantly, PD-L1- and IDO-specific responses were observed irrespective of HLA genotype (Supplementary Table 5).

**Extended Data Fig. 5:**
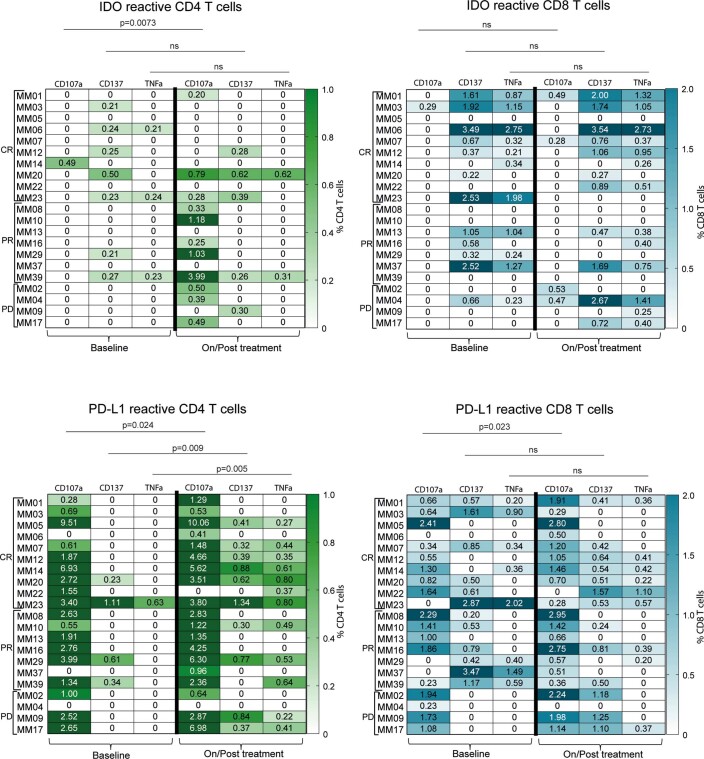
CD4 and CD8 vaccine specific T cell responses in blood. Top: heatmaps of IDO specific CD4 (left) and CD8 (right) T cell responses in PBMCs at baseline and on/post treatment. Bottom: heatmaps of PD-L1 specific CD4 (left) and CD8 (right) T cell responses. Responses quantified by flow cytometry by an increased expression of CD107α, CD137 and TNFα after 8h peptide stimulation. Values represent specific responses after background values have been subtracted (n=21). Statistical analysis were performed using two-sided Wilcoxon matched-paired rank test.

**Extended Data Fig. 6:**
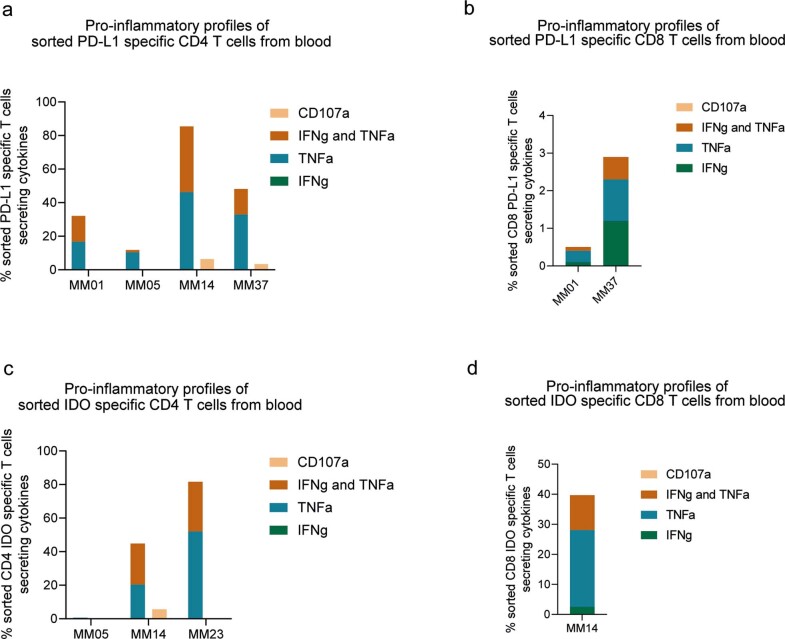
Pro-inflammatory profiles of sorted CD4 and CD8 T cells from blood. **a/b/c/d)** Percentage of in vitro stimulated and sorted PD-L1 and IDO specific CD4 and CD8 T cells secreting cytokines.

To verify the functionality of vaccine-induced T cells, IDO- or PD-L1-specific T cells were isolated and in vitro expanded from PBMCs of five patients. Phenotypic characterization by flow cytometry revealed that the isolated vaccine-specific T cells consisted of both CD4^+^ and CD8^+^ T cells. Also, both IDO- and PD-L1-specific CD4^+^ and CD8^+^ T cells showed pro-inflammatory properties, as they expressed the cytolytic marker CD107a and secreted the cytokines IFN-γ and tumor necrosis factor (TNF)-α (Fig. 3d and Extended Data Fig. 6a–d). Interestingly, we were also able to detect vaccine-specific CD4^+^ and CD8^+^ T cell responses in peripheral blood ex vivo (Extended Data Figs. 4 and 5). We observed a significant increase in the overall percentage of CD107a, CD137 and TNF-α expression in response to peptide stimulation in on- or post-treatment PBMC samples compared to that at baseline, further confirming the expansion and the diverse signature of vaccine-specific T cells (Extended Data Fig. 5).

### Vaccine-specific responses detected in the skin at the vaccination site

To investigate whether vaccine-specific T cells have the potential to migrate to peripheral tissue, delayed-type hypersensitivity (DTH) tests were performed after six cycles of treatment on 15 patients to assess the presence of vaccine-reactive T cells in the skin. Supplementary Table 6 display an overview of skin-infiltrating lymphocyte (SKIL) cultures.

**Extended Data Fig. 7:**
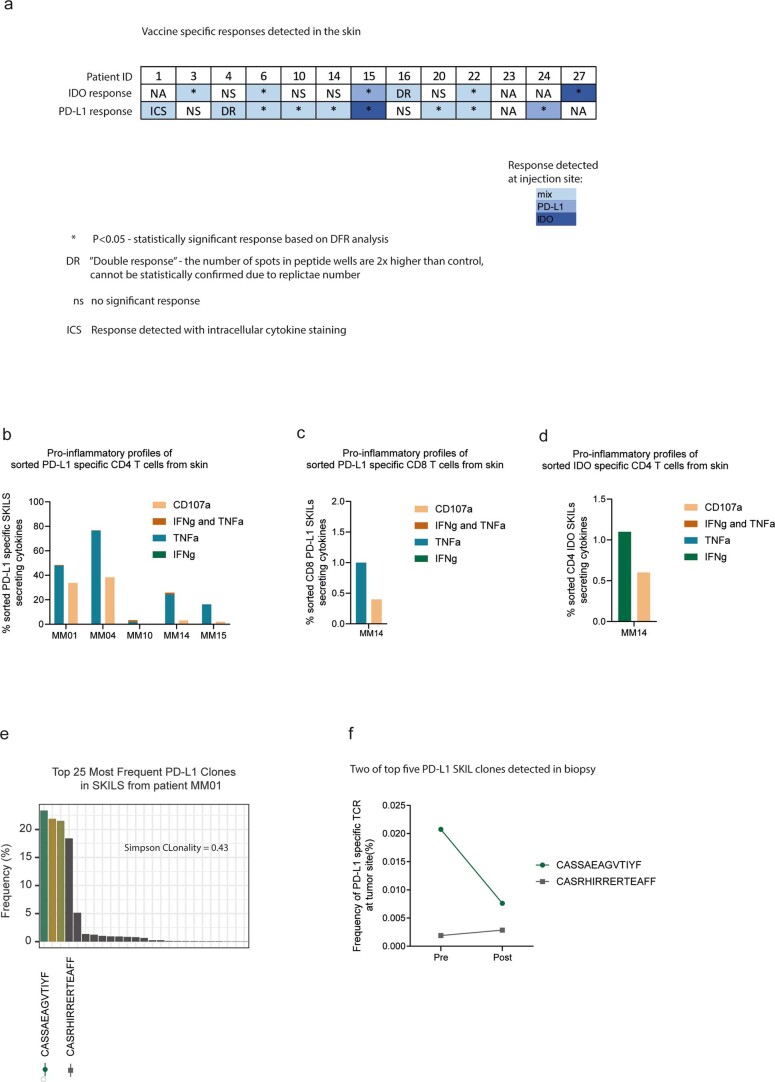
Vaccine specific responses in skin. **a)** IDO and PD-L1 specific T cell responses in SKILs after 6 series of treatment measured by IFN-γ Elispot assay (n=13). SKILs were grown from DTH injection with either IDO peptide, PD-L1 peptide or a mix as presented by different blue colours. * Responses were calculated as the difference between average numbers of spots in wells stimulated with IDO or PD-L1 peptide (triplicates) and corresponding control (DMSO) and statistical analyses of Elispot responses were performed using distribution-free resampling method (Moodie et al.). DR: Not statistically confirmed response due to replicate number but number of spots in peptide wells are two times higher than control wells (DMSO). NS: No significant response and no DR. **b/c/d)** Percentage of cytokine secreting/CD107a+ CD4+ and CD8+ IDO and PD-L1 specific T cells in response to in vitro peptide stimulation by flow cytometry. **e)** Skin infiltrating PD-L1 specific T cell clones also found in biopsy in patient MM01. TCR sequencing was performed on a PD-L1 specific T cell culture generated from DTH area on the lower back injected with PD-L1 peptide on patient MM01. Bars show the frequency of top 25 clones in the culture with which indicates a high Simpson clonality of 0.43. **f)** Tracking the frequency of the top five skin infiltrating PD-L1 specific clones in tumour before and after treatment.

We detected IDO-specific T cells in the skin of six of ten patients and PD-L1-specific T cells in nine of 11 patients (Extended Data Fig. 7a). Intracellular cytokine staining was performed on SKILs from five patients after stimulation with either PD-L1 or IDO peptide. Here, we detected mainly CD4^+^ peptide-reactive T cells that secreted TNF-α and upregulated CD107a, and a minor fraction also secreted IFN-γ. In one patient, we detected CD8^+^ PD-L1-reactive T cells (Extended Data Fig. 7b–d).

### Vaccine-induced T cells specifically recognize target cells

**Fig. 4:**
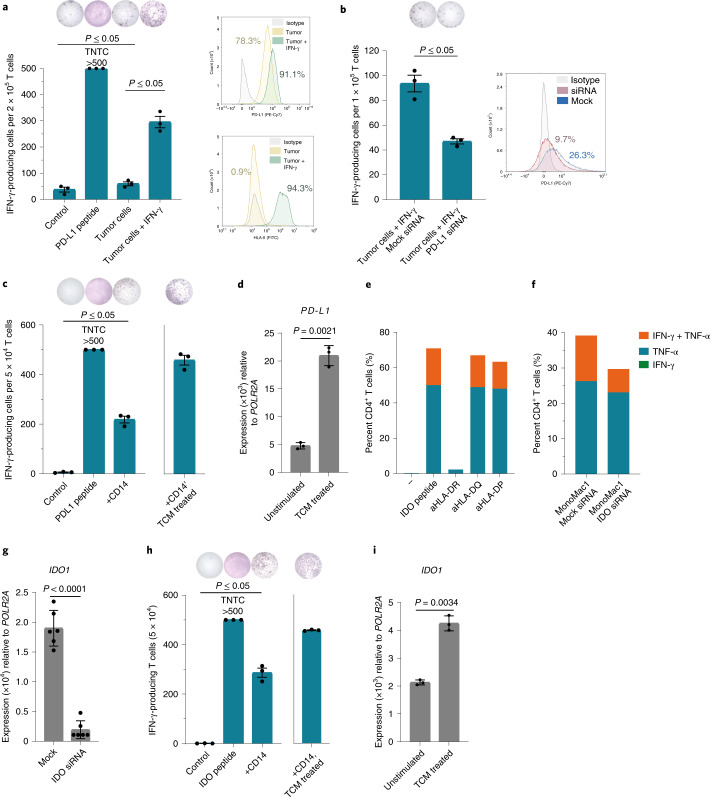
PD-L1- and IDO-specific T cells from vaccinated patients react against PD-L1- and IDO-expressing target cells. **a**, Left, PD-L1-specific T cell culture (MM1636.05) reactivity against PD-L1 peptide or autologous tumor cells in the IFN-γ ELISPOT assay. Tumor cells were either not treated or pretreated with 200 U ml^−1^ IFN-γ for 48 h before the assay. Effector:target (E:T) ratio of 10:1 was used. Right, PD-L1 and HLA-II surface expression on melanoma cells with (green) or without (yellow) pretreatment with IFN-γ compared to an isotype control (gray) as assessed by flow cytometry. **b**, Left, PD-L1-specific T cell (MM1636.05) reactivity in the IFN-γ ELISPOT assay against autologous tumor cells pretreated with IFN-γ (500 U ml^−1^) and transfected with mock or PD-L1 small interfering (si)RNA 24 h after transfection. E:T ratio, 10:1. Right, PD-L1 surface expression on melanoma tumor cells (MM1636.05) assessed by flow cytometry 24 h after transfection with mock (blue) or PD-L1 (red) siRNA compared to the isotype control (gray). **c**, Reactivity of the CD4^+^ PD-L1-specific T cell clone (MM1636.14) against PD-L1 peptide or autologous CD14^+^ cells; E:T ratio, 10:1. CD14^+^ cells were isolated using magnetic bead sorting and used as targets in an ELISPOT assay directly or after pretreatment for 2 d with TCM derived from the autologous tumor cell line. **d**, Quantitative PCR with reverse transcription (RT–qPCR) analysis of *PD-L1* (*CD274*) expression in sorted CD14^+^ cells before and after treatment with autologous TCM for 48 h. **e**, Reactivity of the IDO-specific CD4^+^ T cell clone (MM1636.23) against IDO peptide combined with HLA-DR (L243)-, HLA-DQ (SPV-L3)- or HLA-DP (B7/21)-blocking antibodies (aHLA-DR, aHLA-DQ and aHLA-DP) in an intracellular staining assay (ICS) for IFN-γ and TNF-α production. T cells were incubated with individual blocking antibodies (2 μg ml^−1^) for 30 min before adding IDO peptide. **f**, Reactivity of the IDO-specific CD4^+^ T cell clone (MM1636.23) against the HLA-DR-matched IDO-expressing cell line MonoMac1 transfected with mock or IDO siRNA in an ICS assay, E.T ratio, 4:1. siRNA transfection was performed 48 h before the experiment. **g**, RT–qPCR analysis of *IDO1* expression in MonoMac1 cells 48 h after siRNA transfection. **h**, Reactivity of the CD4^+^ IDO-specific T cell clone (MM1636.14) against IDO peptide or autologous CD14^+^ cells; E:T ratio, 20:1. CD14^+^ cells were isolated using magnetic bead sorting and used as targets in an ELISPOT assay directly or after pretreatment with TCM derived from the autologous tumor cell line. **i**, RT–qPCR analysis of *IDO1* expression in sorted CD14^+^ cells before and after treatment with autologous TCM for 48 h. Bars in RT–qPCR data (**d**,**g**,**i**) represent the mean of three (**d**,**i**) or six (**g**) technical replicates ±s.d.; *P* values were determined by two-tailed parametric *t*-tests. ELISPOT counts (**a**,**b**,**c**,**h**) represent the mean value of three technical replicates ±s.e.m.; response *P* values were determined using the distribution-free resampling (DFR) method. TNTC, too numerous to count.

**Extended Data Fig. 8:**
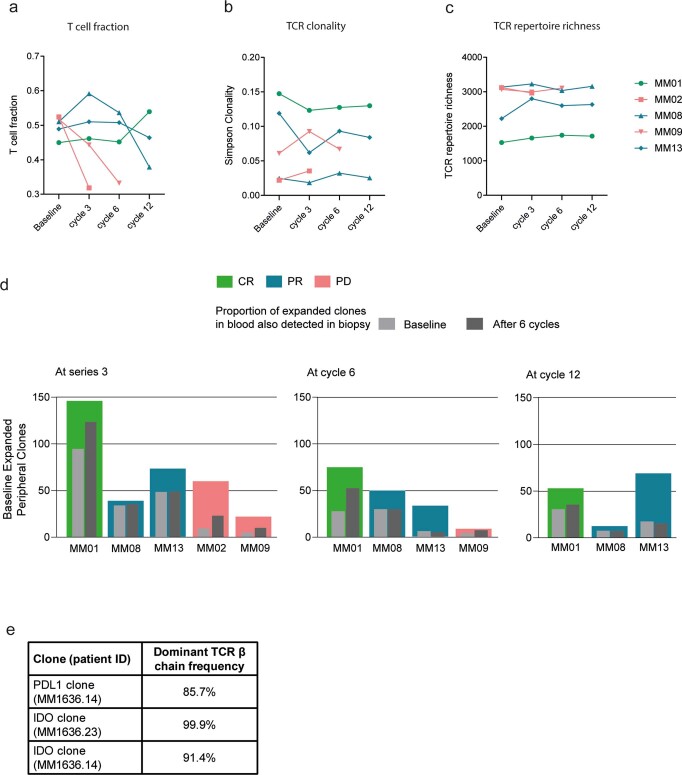
T cell changes in blood after treatment. T cell fraction, TCR clonality and repertoire richness in blood and peripherally expanded TCR clones in both responding and non-responding patients. **a)** T cell fraction in peripheral blood of five patients at baseline, series 3, 6 and 12 by TCR sequencing. T cell fraction was calculated by taking the total number of T cell templates and dividing by the total number of nucleated cells (n=5.) **b)** TCR clonality in peripheral blood of five patients at baseline, series 3, 6 and 12 by TCR sequencing (n=5). Simpson clonality measures how evenly TCR sequences are distributed amongst a set of T cells where 0 indicate even distribution of frequencies and 1 indicate an asymmetric distribution where a few clones dominate. **c)** TCR repertoire richness in peripheral blood of five patients at baseline, series 3, 6 and 12 by TCR sequencing (n=5). TCR repertoire richness report the mean number of unique rearrangements. **d)** Bar-chart chart representing dynamics of the expanded T cell clones. Coloured bars represent peripherally expanded clones in five patients at series 3, series 6 and series 12 as compared to the baseline PBMC samples (n=5). Light gray bar represents peripherally expanded clones that are present in baseline biopsy samples while dark gray bar represents peripherally expanded clones that are present in the post-treatment biopsy (after 6 series). **e)** Frequency of the dominant TCR β chain in clonal PD-L1 and IDO specific cultures as determined by CDR3 sequencing.

To confirm the functionality of vaccine-expanded T cells, vaccine-specific T cell clones (clonal purity was confirmed by T cell receptor (TCR) sequencing) were isolated and expanded from patient PBMCs (Extended Data Fig. 8e). We showed that PD-L1-specific T cells were able to recognize PD-L1^+^ autologous tumor cells in a PD-L1 expression-dependent manner if the cancer cells also expressed HLA-II (Fig. 4a,b). Similarly, an HLA-DR-restricted IDO-specific CD4^+^ T cell clone was able to recognize an HLA-DR-matched IDO-expressing model cell line, MonoMac1, in an IDO-expression-dependent manner (Fig. 4e–g). As previously described, IDO- and PD-L1-specific T cells’ mode of action is not limited to targeting only cancer cells. We were able to show that vaccine-specific T cell clones also reacted against PD-L1- and IDO-expressing autologous immune cells (Fig. 4c,h). To provide myeloid cells with a tumor-associated phenotype we treated isolated CD14^+^ myeloid cells with tumor conditioned medium (TCM) derived from an established autologous tumor cell line. We observed that such TCM-treated CD14^+^ cells had increased expression of PD-L1 and IDO and were effectively recognized by autologous PD-L1- and IDO-specific CD4^+^ T cell clones (Fig. 4c,d,h,i).

### T cell clones in blood and tumors

To track the role of treatment-induced T cell responses, TCR sequencing of the complementarity-determining region 3 (CDR3) was performed on five patients in peripheral blood (baseline and cycles 3, 6 and 12) and paired biopsies. These five patients (MM01, MM02, MM08, MM09 and MM13) were selected due to the availability of material and to investigate a balanced patient group with both responders and non-responders. Due to a limited number of available paired biopsies (either as a consequence of patient refusal or unexpectedly rapid and substantial clinical responses), statistical analysis could not be applied. Details on clinical response are shown in Fig. 2a. Additionally, PBMCs (on treatment) or SKILs were stimulated with the IDO/PD-L1 peptides, and then cytokine-producing T cells were sorted to track vaccine-induced T cells both in the periphery and at the tumor site.

To identify enriched IDO/PD-L1-specific T cell clones, TCR rearrangements in sorted IDO/PD-L1-specific clones and TCR rearrangements in sorted IDO/PD-L1-specific T cell samples were compared to sequences from baseline PBMC samples for each patient. Clonal expansion of vaccine-specific TCR rearrangements from samples on vaccination were then tracked using a differential abundance framework. Cumulative IDO/PD-L1-specific T cell frequencies were tracked in post-treatment samples.

We found no relation between clinical response and the enrichment of vaccine-specific clones, but an increase in IDO/PD-L1-specific T cell clones was observed at different time points in the periphery in all five patients (Extended Data Fig. 3c).

We next investigated overall changes in the T cell repertoire in the blood. A modest increase in the peripheral T cell fraction was observed in the three responding patients at cycle 3, while the two non-responding patients had a clear decrease in T cell fraction (Extended Data Fig. 8a). We thereafter investigated TCR clonality and TCR repertoire richness, exploring the proportion of abundant clones and the number of unique rearrangements, respectively. A decreasing peripheral Simpson clonality and increasing TCR repertoire richness was observed in responding patients at cycle 3, which might indicate tumor trafficking upon treatment. The opposite pattern was observed in non-responding patients (Extended Data Fig. 8b,c).

Peripherally expanded clones were associated with tumors and persisted until cycle 12 (latest time point analyzed). The largest peripheral expansion was observed at cycle 3, with the most significant increase observed in patient MM01 (CR). Responding patients had a larger fraction of peripherally expanded clones that were also found in tumors compared to that of non-responders. By tracking peripherally expanded clones detected at the tumor site, we observed that patient MM01 had a substantial increase after treatment, indicating tumor trafficking of peripheral expanded clones (Extended Data Fig. 8d).

### Influx at the tumor site of vaccine-enriched T cell clones

Given the observation of increased T cell fraction and enrichment of IDO- and PD-L1-specific clones in the blood after treatment, we investigated whether the same trend was observed at the tumor site.

**Fig. 5:**
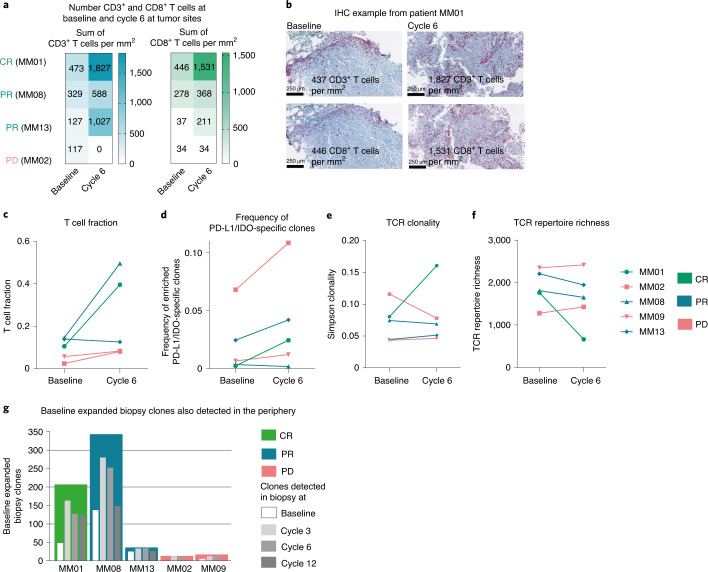
Changes in the TME after treatment. Number of CD3^+^ and CD8^+^ T cells, TCR fraction, TCR clonality, TCR repertoire, biopsy expanded TCR clones and enrichment of IDO- and PD-L1-specific T cells at the tumor site. **a**, Number of CD3^+^ and CD8^+^ T cells per mm^2^ (sum in the validated area) at the tumor site detected by IHC of paired biopsies from four patients. **b**, Example of IHC of CD3^+^ and CD8^+^ T cells at the tumor site before and after treatment (cells per mm^2^) in one patient (MM01). **c**, T cell fraction at the tumor site at baseline and cycle 6 by TCR sequencing. The T cell fraction was calculated by taking the total number of T cell templates and dividing by the total number of nucleated cells. **d**, Tracking of vaccine-associated clones at baseline and cycle 6 in tumor biopsies. Cumulative frequencies of IDO and PD-L1 vaccine-specific TCR rearrangements are represented. **e**,**f**, TCR clonality and TCR repertoire richness in five patients at the tumor site at baseline and cycle 6. Simpson clonality measures how evenly TCR sequences are distributed among a set of T cells, where 0 indicates an even distribution of frequencies and 1 indicates an asymmetric distribution in which a few clones dominate. TCR repertoire richness reports the mean number of unique rearrangements. **g**, Bar chart representing baseline expanded biopsy clones from five patients (colored bars) and the detection of biopsy expanded clones also found in the blood at baseline and series 3, 6 and 12 (white and gray bars) by TCR sequencing.

Both TCR sequencing and immunohistochemistry (IHC) of paired biopsies from the five patients described above showed an increase in the T cell fraction with an influx of CD3^+^ and CD8^+^ T cells after treatment in the three responding patients (Fig. 5a–c). IHC was not possible for patient MM09 due to tissue loss.

We thereafter investigated whether some of the IDO/PD-L1 vaccine-linked T cells were present at the tumor site. Vaccine-associated clones were tracked as the combined frequency of IDO- and PD-L1-specific T cell rearrangements. In biopsies, the frequencies at cycle 6 were compared to those at baseline and showed an increase in vaccine-specific T cells in four of five patients, irrespective of clinical response (Fig. 5d). TCR sequencing of PD-L1-specific SKILs (a more specific culture than IDO/PD-L1-specific isolations derived from PBMCs on vaccination) and paired biopsies showed that two of the top five PD-L1-specific SKIL clones were present at the tumor site both before and after treatment (Extended Data Fig. 7e,f).

With a focus on the more abundant T cell clones, we investigated overall TCR clonality at the tumor site before and after treatment. In addition, we explored the number of unique TCR rearrangements, dissecting the lower-frequency clones. Patient MM01 had a significant increase in TCR clonality and a decrease in repertoire richness at the tumor site after therapy, indicating a focused tumor repertoire response of selected clones. All three responding patients had a decrease in TCR repertoire richness, which again might indicate a focused tumor response (Fig. 5e,f).

Deeper analyses showed that the T cell clones that expanded at the tumor site after therapy were also present in the blood at baseline and increased significantly after treatment in four of five patients. The highest proportion was detected early at cycle 3. These data again support trafficking of peripherally expanded clones to the tumor site and could indicate that the T cell response to treatment is derived from pre-existing peripherally tumor-associated T cells (Fig. 5g).

### Signs of treatment-induced inflammation in the TME

**Extended Data Fig. 9:**
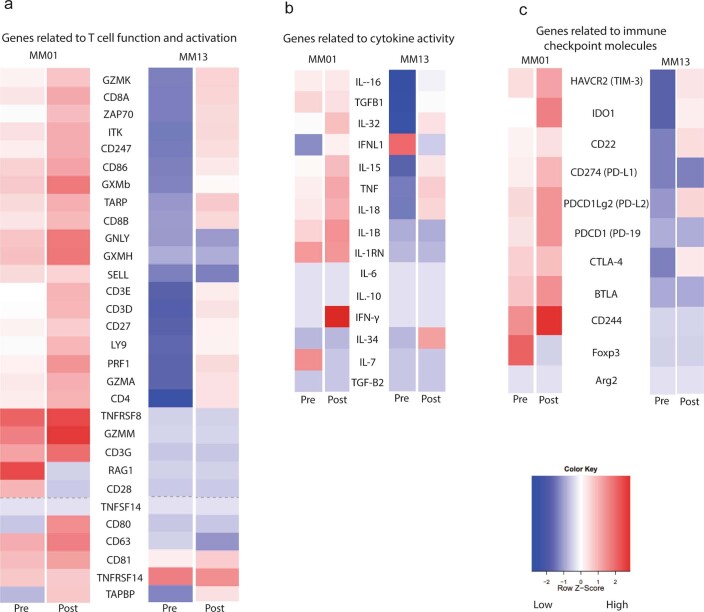
Profiling of genes relevant to T cell activation, cytokines and exhaustion markers on pre- and post-treatment biopsies in two patients. **a)** RNA expression profiling of genes related to T cell activation was performed using NanoString nCounter (n=2). **b)** RNA expression profiling of genes related to cytokine activity was performed using NanoString nCounter (n=2). **c)** RNA expression profiling of genes related to checkpoint inhibitors was performed using NanoString nCounter (n=2).

To dissect changes in the TME induced by T cell influx upon treatment in responding patients, RNA gene expression analyses using the nCounter PanCancer Immune Profiling Panel from NanoString were performed on paired biopsies from two responding patients (MM01 and MM13). Expression of genes related to adaptive immunity such as T cell activation, effector functions (genes encoding IFN-γ, TNF-α, IL-15 and IL-18) and cytotoxicity was increased in post-treatment biopsies (Extended Data Fig. 9a,b). Also, expression of genes related to checkpoint inhibitors such as those encoding T cell immunoglobulin and mucin-domain containing-3 (TIM-3), IDO, PD-L1, PD-L2, PD-1 and CTLA-4 increased after treatment, indicating activation of immune cells in the TME (Extended Data Fig. 9c).

**Extended Data Fig. 10:**
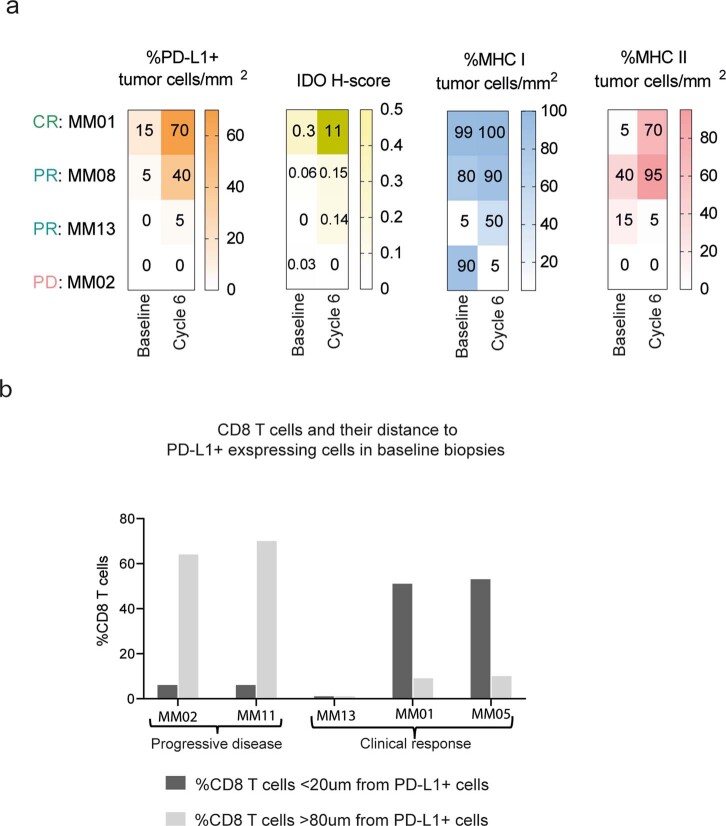
Treatment induced upregulation of PD-L1, IDO, MHC I and MHC II and distance between CD8 T cells and PD-L1 expressing cells. **a)** IHC on 4 paired biopsies stained for CD3 and CD8 T cells, PD-L1, MHC I and MHC II on tumor cells/mm^2^ (sum in the validated area) and IDO H-score (from 0 to 300). H-score is the expression of IDO on both immune and tumor cells: The score is obtained by the formula: 3 x percentage of cells with a strong staining + 2 x percentage of cells with a moderate staining + percentage of cells with a weak staining (n=4). **b)** Distance in µm between CD8+ T cells and PD-L1+ stained cells on five baseline biopsies detected by IHC (n=5).

Additionally, IHC of paired biopsies from four patients (MM01, MM02, MM05 and MM13) showed an upregulation of PD-L1, IDO, MHC-I and MHC-II on tumor cells, indicating a treatment-induced pro-inflammatory response in the three responding patients, except for a decrease in MHC-II expression in patient MM13. By contrast, the non-responding patient MM02 had a reduction in T cell numbers present in the tumor after treatment and no expression of PD-L1, IDO or MHC-II and, interestingly, total loss of MHC-I, demonstrating tumor immune escape (Extended Data Fig. 10a).

CD8^+^ T cells and their distance (µm) to PD-L1-expressing cells in baseline biopsies from five patients was investigated by IHC. Except for patient MM13 (PR), distance and clinical responses were associated: the two responders had reduced distance (<20 µm) between cells expressing these markers compared to non-responding patients (>80 µm). This observation indicates that responding patients not only have a higher intratumoral infiltration of CD8^+^ T cells, but that these cells can surround and attack PD-L1-expressing immune cells and tumor cells (Extended Data Fig. 10b).

## Discussion

In this clinical trial, MM1636, 30 patients with metastatic melanoma were treated with a first-in-class immunomodulatory IDO/PD-L1-targeting peptide vaccine combined with nivolumab. The treatment led to an unprecedented high ORR of 80%, with 43% of patients reaching a CR, and a striking mPFS of 26 months (95% CI, 15.4–69 months) was reached. The vaccine represents a new treatment strategy to activate specific T cells that target cells contributing to immune suppression (including tumor cells), positively modulating the TME by inducing local inflammation. Indeed, we show that vaccine-specific T cells isolated and expanded from vaccinated patients recognize not only tumor cells in a target- and HLA-restricted manner but also myeloid cells polarized toward a tumor-associated phenotype. Hence, myeloid cells become targets for vaccine-activated T cells when they have a tumor-associated phenotype in the TME. These phenomena may further induce checkpoint molecules and rewire the TME toward an increasingly aPD1-permissive state.

A drawback of our study is the single-center nonrandomized setup. Comparison between trials or between patients in trials and real-world patients is problematic due to multiple factors, such as period of conduction and other therapies available at the different periods of time. Nevertheless, the rate of investigator-assessed ORR in the phase 3 trial CheckMate 067 was 43.7% in the nivolumab monotherapy group and 57% in the nivolumab and ipilimumab group. CRs occurred in 8.9% and 11.5% of patients, respectively^18^. The mPFS of 26 months (95% CI, 15.4–69 months) in this trial is more than twice as long as that for patients treated with nivolumab and ipilimumab in CheckMate 067, for which an mPFS of 11.5 months (95% CI, 8.7–19.3 months) was reached.

Patient baseline characteristics were in general comparable to those of patients with MM who have been treated in CheckMate 067, although patients in MM1636 were older (mean age, 70 years) and a larger fraction were positive for PD-L1 (57%)^3,18,22^. Among patients with PD-L1-negative tumors in MM1636, an ORR of 61.5% was still reached, which would be expected to be around 33% for first-line nivolumab monotherapy^23^. Some studies suggest that older patients might have a tendency to respond better to aPD1 therapy; however, this is still debated^24–27^.

To address potential trial bias and the nonrandomized setup, patients in MM1636 were matched for age, performance status, sex, M stage, LDH level, PD-L1 status and *BRAF* status with a historical control group from the DAMMED, who were treated contemporarily (2015–2019) with aPD1 monotherapy as standard of care^17^. We found a significantly higher ORR and CRR in MM1636 compared to those of matched patients, who had an ORR of 43% and a CRR of 13%, comparable to patients treated in CheckMate 067. Restrictions of the synthetic control group are of course that it is partially historic and patient selection outside matching criteria cannot be ruled out^28^.

Numerous contemporary clinical trials are exploring the combination of aPD1 therapy with other immunomodulating agents for advanced melanoma.

Talimogene laherparepvec, an oncolytic virus, is approved by the Food and Drug Administration and the European Medicines Agency to treat advanced melanoma. A small phase 1b trial with 21 patients (MASTERKEY-265) combined talimogene laherparepvec and pembrolizumab to treat patients with advanced unresectable melanoma and reached an ORR of 62% and a CR of 33%^29,30^. Seventy-one percent of the patients in this trial had an M stage below M1c; this number was 40% in our trial. Furthermore, mainly patients with an M stage below M1c responded to treatment, which was not the case in MM1636. Results from a large randomized phase 3 trial are awaited (KEYNOTE-034).

Epacadostat, an IDO inhibitor, was tested in combination with pembrolizumab in a nonrandomized phase 2 trial in 40 aPD1 treatment-naive patients with MM with promising results, reaching an ORR of 62%. Unfortunately, the phase 3 trial showed no indication that epacadostat provided improvement in PFS and OS^31^. Limitations of the phase 3 trial were the sparse information on pharmacodynamics as well as biomarker evaluation to improve the design. The IDO/PD-L1 vaccine is different from epacadostat, as it is not an IDO inhibitor but targets IDO- and PD-L1-expressing cells. Similar vaccines administered as monotherapy induced objective responses in lung cancer and basal cell carcinoma, while epacadostat as monotherapy in 52 patients resulted in no responses^32,33^.

Sahin et al. recently published encouraging data from a first-in-human trial, in which a vaccine containing liposomal RNA targeting four unmutated tumor-associated antigens (NY-ESO-1, MAGE-A3, tyrosinase and TPTE) was administered alone or in combination with nivolumab in patients with advanced melanoma. Responses were observed in both the monotherapy group as well as the combination group in checkpoint inhibitor-experienced patients, suggesting the efficacy of non-mutant shared tumor antigen vaccines^34^.

The overall safety and tolerability findings are comparable to those of aPD1 monotherapy. Injection site reactions were exclusive to the vaccine. However, these side effects were transient and mild in most patients and most likely due to the adjuvant Montanide. IDO- and PD-L1-specific CD8^+^ and CD4^+^ T cells exist among peripheral blood lymphocytes in healthy donors^35–38^ and expand in response to pro-inflammatory stimuli^35,39^. Furthermore, both IDO and PD-L1 are induced in cells as a counter response to the inflammatory response. This provides a mechanism that ensures immune homeostasis, which keeps IDO/PD-L1-specific T cells in check; therefore, the risk of triggering autoimmune-related adverse events by vaccination appears to be minimal. This was confirmed in the first clinical trials of IDO and PD-L1 vaccination (NCT01543464 (refs. ^33,40^) and NCT03042793 (ref. ^41^)).

The spontaneous (baseline) immune response to the vaccines observed in the current study is in agreement with our previous observation in various patients with cancer^35–38^. Numerous vaccine-induced changes in the blood and at the tumor site were observed. Peripheral IDO- and/or PD-L1-specific T cells were detected in vitro in a modified ELISPOT in over 93% of patients on vaccination, unrelated to patient HLA type. Immune responses were persistent in patients who surpassed follow-up at data cutoff and were still detectable up to 6 months after the last vaccine, suggesting induction of memory T cells. We did not observe a correlation between vaccine-induced responses in blood and clinical responses. However, the detection of a highly significant increase in vaccine-specific T cell numbers after vaccination in almost all patients together with the very low number of patients with PD makes it difficult to expect such a correlation, especially because other aspects should be taken into account (for example, the loss of class I expression on tumors cells in patient MM02 with PD after vaccination). Frequencies of peripheral T cells induced against PD-L1 were overall higher than those against IDO. Importantly, however, we also observed that it was not the same patients who responded strongly to both IDO and PD-L1, nor did patients react with a similar response pattern to the two antigens; that is, different time points of response were observed. This suggests that each component of the vaccine plays different roles in the ongoing immune response in patients. The goal of IDO/PD-L1 vaccination was to modulate the TME to increase responsiveness to aPD1 therapy, as we have observed in animal models, in which immune conversion is demonstrated in the TME with an increased influx of T cells. Targeting both IDO and PD-L1 together enables synergy, as the TME is known to use different immune escape mechanisms and IDO and PD-L1 are often overexpressed by different cellular compartments. Ex vivo ELISPOT and flow cytometry assays confirmed the induction of immune response toward both epitopes and the higher immunogenicity of the PD-L1 epitope, although because of lower sensitivity failed to detect some of the responses. TCR sequencing of five patients confirmed enrichment of IDO/PD-L1-specific T cell clones in the blood at different time points after treatment. Furthermore, an increase in enriched IDO/PD-L1-specific clones was observed in four of five patients at the tumor site after treatment, irrespective of clinical response.

Phenotypic characterization showed that vaccine-specific T cells, which were expanded in vitro with interleukin (IL)-2 from the blood of vaccinated individuals, were both CD4^+^ T cells and CD8^+^ T cells. This was confirmed by ex vivo phenotype description of vaccine-activated T cells. Vaccine-specific T cells expressed CD107a and CD137 and produced IFN-γ and TNF-α upon stimulation with the cognate target, indicating their cytolytic capacity.

Overall, the data from immune monitoring supports the importance of both antigens in the generation of the frequent clinical responses in the study.

Despite a limited number of paired biopsies (due to either patient refusal or the fact that a large fraction of responding patients had no assessable tumors after six cycles), we observed trends indicating treatment-induced general T cell influx in responding patients. It was shown that proliferation of CD8^+^ T cells in the tumor after aPD1 treatment is associated with radiographic reduction in tumor size^42^. Additionally, we showed that a large proportion of expanded peripheral TCR clones were associated with tumors and the most considerable amount of clonal expansion was observed early, at cycle 3. For the patient with a CR included in the TCR sequencing analyses (MM01), the number of peripheral expanded clones present at the tumor site increased after treatment compared to that at baseline, indicating tumor trafficking of peripheral expanded clones.

Gene expression analyses (two paired biopsies) and IHC (five paired biopsies) further demonstrated that the combination treatment induced a pro-inflammatory TME in responding patients with signs of T cell activation and cytotoxicity and increased cytokine activity. This may lead to further upregulation of IDO, PD-L1, MHC-I and MHC-II on tumor cells, leading to more treatment targets. It was shown that, following vaccination with a cancer vaccine, PD-L1 expression is increased on tumor cells due to recruitment of tumor-specific T cells and upregulation of adaptive immune resistance pathways in the TME^43^. Treatment with nivolumab monotherapy enhances PD-L1 expression, and it is therefore problematic to discriminate the effect of the vaccine as compared to that of nivolumab^44^.

IDO- and PD-L1-specific pro-inflammatory effector T cells were hypothesized to counteract the functions of IDO- and PD-L1-expressing immune-suppressive cells as a means to keep the immune balance between immune activation and inhibition^45^. However, because the expression of these molecules is induced as a counter-regulatory response to inflammatory mediators such as IFNs, they can also be expressed by, for example, activated myeloid cells or T cells. Thus, activation of PD-L1-specific T cells may result in depletion of activated T cells in the tumor and other sites. Importantly, it was recently described that PD-L1^+^ T cells mainly have tolerogenic effects on tumor immunity and exert tumor-promoting properties, suggesting that targeting this immune population is indeed also beneficial^46^. We have previously investigated the effect of activating PD-L1-specific T cells in vitro and in vivo and found that, overall, they supported the effector phase of an immune response by removing PD-L1-expressing regulatory immune cells that inhibit PD-1^+^ effector T cells^47,48^. The major role of the PD-1 pathway is believed to be the regulation of effector T cell responses. Thus, this protective pathway is more important after activation, rather than at the initial T cell-activation stage^49^. Accordingly, the presence of PD-L1-specific T cells during the activation phase of an immune response may not increase or support a pro-inflammatory immune response due to the expression of PD-L1 on potent antigen-presenting cells or on the T cells themselves^14^. Thus, the overall effects of PD-L1-specific T cells may vary depending on the expression of both PD-1 and PD-L1, that is, due to the microenvironment and the state of the immune response.

In conclusion, here we report an impressive response rate, CR rate and mPFS for a first-in-class immune-modulating vaccine combined with nivolumab. This may be a first step toward a new treatment strategy for patients with MM. Limitations are the low number of patients treated at a single institution and the lack of a randomized design with aPD1 monotherapy as comparator. Studies in aPD1 therapy-resistant or -refractory melanoma are ongoing as well as biomarker analysis for selecting patients at a higher likelihood to benefit from the combination versus aPD1 monotherapy. A larger randomized trial will be essential to validate these findings and determine the specific contribution of the vaccine to clinical responses and changes in the TME. In December 2020, the Food and Drug Administration granted breakthrough therapy designation for the IO102/IO103 vaccine combined with aPD1 therapy in metastatic melanoma based on data from the MM1636 trial.

## Methods

### Trial design and treatment plan

MM1636 is an investigator-initiated, nonrandomized, open-label, single-center phase 1/2 study. All patients were treated at the Department of Oncology, Herlev and Gentofte Hospital, University of Copenhagen, Herlev, Denmark. The study was conducted according to the Declaration of Helsinki and Good Clinical Practice (GCP) and monitored by the GCP unit in Copenhagen, Denmark. The protocol was approved by the ethical committee of the Capital region of Denmark (H-17000988), the Danish Medical Agencies (2017011073) and the Capital Region of Denmark Data Unit (P-2019-172). The study was registered at https://clinicaltrials.gov/ under the identifier NCT03047928 and at the EudraCT (no. 2016-0004527-23).

This study initially aimed to include 30 aPD1 treatment-naive patients with MM. An amendment with the addition of two other cohorts with ten patients in each cohort was made to evaluate immune responses and clinical efficacy in aPD1 therapy-resistant patients (cohort B, de novo resistance and cohort C, acquired resistance) for a total of 50 patients. The amendment with cohorts B and C was approved at the inclusion of 18 patients in cohort A. The trial is still including patients in cohorts B and C. This article reports results from cohort A.

The first six patients were treated as for phase 1, evaluating for safety and tolerability before the remaining 24 patients were included in phase 2.

The IDO/PD-L1 vaccine was administered subcutaneously biweekly for the first 10 weeks and thereafter every 4 weeks for approximately 9 months. A maximum of 15 vaccines were administered. Nivolumab was administered according to the approved label (3 mg per kg biweekly) for 24 cycles. The 15th vaccine was administered together with the 24th nivolumab cycle of 3 mg per kg, and responding patients thereafter continued nivolumab monotherapy every 4th week (6 mg per kg) as a standard of care after investigator assessment. Treatment with nivolumab was discontinued at the maximum benefit (assessed by the investigator), after a maximum of 2 years of therapy, at progression or due to severe adverse events (Extended Data Fig. 1b, treatment plan).

### Vaccine composition

Each vaccine was composed of 100 µg IO102, a 21-amino-acid peptide (DTLLKALLEIASCLEKALQVF) from IDO, and 100 µg IO103, a 19-amino-acid peptide (FMTYWHLLNAFTVTVPKDL) from the signal peptide of PD-L1 (PolyPeptide Laboratories). Peptides were dissolved separately in 50 µl DMSO, filtered for sterility and frozen at −20 °C (NUNC CyroTubes CryoLine System Internal Thread, Sigma-Aldrich). At <24 h before administration, peptides were thawed. The PD-L1 peptide was diluted in 400 µl sterile water and, immediately before injection, mixed with the IDO peptide solution and 500 µl Montanide ISA-51 (SEPPIC) to achieve a total volume of 1 ml.

### Patients

Patients above 18 years of age with locally advanced or stage 4 melanoma according to the AJCC (seventh edition), at least one measurable lesion according to RECIST 1.1 and an ECOG PS of 0–1 were eligible. The main exclusion criteria were prior treatment with aPD1 therapy, CNS metastases >1 cm, severe comorbidities and active autoimmune disease. Enrollment was not restricted to PD-L1 status, but it was known before inclusion. Patients were included after informed consent.

Patients MM42 and MM20 have confirmed their approval of PET–CT images and clinical images that have been included in the article.

### Key study assessments

Safety and tolerability were evaluated based on changes in clinical laboratory analyses and reported adverse events. Adverse events were assessed according to Common Terminology Criteria for Adverse Events (version 5.0) and were graded from 1 to 5 for all treated patients up to 6 months after the last dose of the IDO/PD-L1 vaccine.

Clinical efficacy was assessed using FDG PET–CT scans before treatment and every 3rd month until progression. Objective responses were categorized into CR, PR, stable disease or PD according to RECIST version 1.1.

Clinical data were collected at CCIT-DK in the eCRF program OpenClinica version 1.0 and in Microsoft Excel version 2002 on a secure server.

Blood samples for immunologic analyses were collected before treatment, before the third cycle, after the sixth, 12th, 18th and 24th cycles (on vaccination) and 3 and 6 months after the last vaccine.

Two to three tumor needle biopsies (1.2 mm) were collected at baseline and after six cycles from the same tumor site, when accessible, to evaluate immune responses at the tumor site.

DTH skin tests were performed and punch biopsies were taken from the DTH area after cycle 6 for the evaluation of SKILs reactive to PD-L1 and IDO (Supplementary Fig. 1, treatment plan).

### Statistical analysis of clinical outcome

Survival curves were computed in GraphPad Prism software version 9.0.0 according to Kaplan–Meier method. Median follow-up time of enrollment was calculated using the reverse Kaplan–Meier method, also in GraphPad Prism software version 9.0.0.

For binary outcomes, 95% two-sided CIs were constructed using the Clopper–Pearson method, also in GraphPad version 9.0.0.

An independent board of certified and experienced oncoradiologists performed an external review to evaluate clinical response to address the potential bias of investigator site review. The external review took place at Rigshospitalet, Copenhagen University Hospital. This hospital did not participate in the MM1636 trial, and the external reviewer had no prior knowledge about the clinical trial or the trial therapy. Only PET–CT images were accessed, and arrows indicating target or non-target lesions appeared on baseline images as the only additional information.

To address potential trial bias regarding treatment effect, we matched patients in MM1636 with patients from the DAMMED, a population-based database that retrospectively collects data on patients with metastatic melanoma in Denmark. Here, data from 938 patients treated with aPD1 monotherapy contemporaneously (January 2015–October 2019) were extracted. Two hundred and eighteen of these patients were eligible for comparison and matching (all parameters available) (Supplementary Table 1), and 74 patients from the DAMMED were found to match. Patients were matched for age (≤70 years, >70 years), sex, LDH levels (normal, elevated), M stage (M1a, M1b, M1c), *BRAF* status (wild type, mutated) and PD-L1 status (<1%, ≥1%). An exact matching algorithm was used in which patients in MM1636 were matched with patients from the DAMMED with the same combination of variables. Twenty-nine patients from MM1636 were matched with exact combinations of the six variables. One patient could not be matched. To secure balance of the calculations, control patients were weighted according to the number of patients for each MM1636 patient. Estimates for treatment effects were calculated by weighted logistic regression analyses and weighted Cox proportional hazard model. The R package ‘Matchlt’ was used for matching patients.

As the method chosen for matching control patients to protocol patients, a weighted binary logistic regression model was used for comparing response rates in the two matched cohorts. Odds ratios and response rates, including their corresponding 95% CIs, were extracted from the regression models. All *P* values were two sided, and *P* values below 0.05 were considered statistically significant. SAS version 9.4M5 was used for the weighted logistic regression models.

### Processing of peripheral blood mononuclear cells

Peripheral blood was collected from all patients in heparinized tubes and was processed within 4 h. In brief, PBMCs were isolated using Lymphoprep (Medinor) separation. PBMCs were counted on a Sysmex XP-300 analyzer and frozen in Human AB Serum (Sigma-Aldrich, H4522-100ML) with 10% DMSO using controlled-rate freezing (CoolCell, BioCision) in a −80 °C freezer and were moved the next day to a freezer at −140 °C until further processing.

### Needle biopsies at baseline and after the sixth vaccine

Two to three needle biopsies (1.2 mm) were taken at baseline and after the sixth cycle of treatment when assessable from the same tumor lesion.

One fragment was fixed with formalin and embedded in paraffin (FFPE); one to two fragments were used for expanding tumor-infiltrating T cells and establishing autologous tumor cell lines.

### Delayed-type hypersensitivity and generation of skin-infiltrating lymphocytes

After six cycles of treatment, we performed intradermal injections of vaccine components without adjuvant and one control injection containing DMSO without peptide. Patients were injected with either a mixture of both IDO and PD-L1 peptides at all three injection sites or PD-L1 peptide, IDO peptide or a mixture of the two at the injection sites, respectively (Supplementary Table 6). Eight hours after injection, punch biopsies were resected from the three sites where the peptide was injected and transported immediately to the laboratory and cut into fragments of 1–2 mm^3^.

SKILs were expanded to establish ‘young SKILs’ in CM medium consisting of RPMI 1640 with GlutaMAX, 25 mM HEPES, pH 7.2 (Gibco, 72400-021), IL-2 (100 or 6,000 IU ml^−1^) (Proleukin Novartis, 004184), 10% heat-inactivated Human AB Serum (Sigma-Aldrich, H4522-100ML), 100 U ml^−1^ penicillin, 1.25 µg ml^−1^ Fungizone (Bristol Myers Squibb, 49182) and 100 µg ml^−1^ streptomycin (Gibco, 15140-122). Half of the medium was replaced three times per week.

Young SKILs from samples derived from IDO peptide, PD-L1 peptide and the mixture injection sites were further expanded in a small-scale version of the 14-d rapid expansion protocol as previously described^51^.

### Quantification of specific T cells in blood by ELISPOT

For enumeration of vaccine-specific T cells in the peripheral blood, PBMCs from patients were stimulated with IDO or PD-L1 peptide in the presence of a low dose of IL-2 (120 U ml^−1^) for 7–13 d before being used in IFN-γ ELISPOT assays with 2.5–3.2 × 10^5^ cells per well.

Briefly, cells were placed in a 96-well PVDF membrane-bottomed ELISPOT plate (MultiScreen MSIPN4W50, Millipore) precoated with IFN-γ-capture antibody (clone 1-D1K, Mabtech). Diluted IDO or PD-L1 peptide stocks in DMSO were added at 5 µM; an equivalent amount of DMSO was added to control wells. PBMCs from each patient were set up in duplicate or triplicate for peptide and control stimulations. Cells were incubated in ELISPOT plates in the presence of the peptide for 16–18 h, after which they were washed off, and biotinylated secondary antibody (anti-human IFN-γ mAB, clone 7-B6-1, Mabtech) was added. After 2 h of incubation, unbound secondary antibody was washed off, and streptavidin-conjugated alkaline phosphatase (Mabtech) was added for 1 h. Next, unbound streptavidin-conjugated enzyme was washed off, and the assay was developed by adding BCIP/NBT substrate (Mabtech). ELISPOT plates were analyzed on the CTL ImmunoSpot S6 Ultimate V analyzer using ImmunoSpot software version 5.1. Responses were calculated as the difference between the average numbers of spots in wells stimulated with IDO or PD-L1 peptide and those from corresponding control wells.

For detection of IDO and PD-L1 peptide responses ex vivo, PBMCs were thawed and rested overnight in medium containing DNase I (1 μg ml^−1^, Sigma-Aldrich, 11284932001) before being used in IFN-γ ELISPOT assays as described above. A total of 6–9 × 10^5^ PBMCs per well were seeded. Statistical analysis of all ELISPOT responses was performed using the DFR method as described by Moodie et al. using RStudio software (RStudio Team, 2016, http://www.rstudio.com/)^50^.

Vaccine-specific ELISPOT responses were defined as true if the difference between the spot count in control and peptide-stimulated wells was statistically significant according to DFR statistical analysis or, for samples performed in duplicate, if the spot count in peptide-stimulated wells was at least 2× the spot count in control wells^50^.

### Cancer cell lines and tumor conditioned medium

Autologous melanoma cell lines were established from needle biopsies. Briefly, biopsies were chopped into small fragments and seeded in 24-well cultures in RPMI 1640 with GlutaMAX, 25 mM HEPES, pH 7.2 (Gibco, 72400-021), 10% heat-inactivated FBS (Life Technologies, 10500064), 100 U ml^−1^ penicillin, 1.25 µg ml^−1^ Fungizone (Bristol Myers Squibb, 49182) and 100 µg ml^−1^ streptomycin (Gibco, 15140-122). Established adherent melanoma tumor cell lines were cryopreserved at −140 °C in freezing medium containing FBS with 10% DMSO. PD-L1 and HLA-II expression on established tumor cell lines was assessed by flow cytometry staining with anti-PD-L1–PE-Cy7 (1:23 dilution, clone M1h1 (RUO), BD, 558017) and anti-HLA-II–FITC (1:23 dilution, clone Tu39 (RUO), BD, 555558) antibodies.

To obtain TCM, established tumor cell lines were cultured in 175-cm^2^ Nunc cell culture flasks until 80–90% confluency was reached. The culture medium was then replaced with 20 ml fresh X-VIVO 15 with Gentamicin and Phenol Red (Lonza, BE02-060Q), medium with 5% heat-inactivated Human AB Serum (Sigma-Aldrich, H4522-100ML). After 24 h of incubation, TCM was collected and centrifuged to remove any resuspended cells, after which TCM was aliquoted, frozen and stored at −80 °C.

The acute monocytic leukemia cell line MonoMac1 was obtained from the DSMZ—German Collection of Microorganisms and Cell Cultures (ACC 252) and cultured in RPMI 1640 with GlutaMAX, 25 mM HEPES, pH 7.2 (Gibco, 72400-021) and 10% heat-inactivated FBS.

### Isolation of autologous myeloid cells

Autologous CD14^+^ cells were sorted from freshly thawed PBMCs using a magnetic bead-separation kit (Miltenyi Biotec, 130-050-201) according to the manufacturer’s instructions. Isolated CD14^+^ cells were used as targets in the IFN-γ ELISPOT assay directly after sorting or differentiated in vitro into tumor-associated macrophages by culturing with 1 ml fresh X-VIVO 15 medium with Gentamicin and Phenol Red (Lonza, BE02-060Q) and 5% heat-inactivated Human AB Serum, supplemented with 1 ml autologous TCM in 24-well plates for 2 d.

### Quantification of vaccine-specific T cells from DTH biopsy sites by ELISPOT

SKILs expanded in vitro were rested in medium without IL-2 overnight before being used in the IFN-γ ELISPOT assay as described above to evaluate reactivity of skin-infiltrating T cells.

### Generation of IDO- and PD-L1-specific T cell cultures from PBMCs or SKILs

IDO- or PD-L1-specific T cells were isolated from peptide-stimulated in vitro PBMC cultures on days 14–15 after stimulation or from SKIL cultures expanded in vitro. For specific T cell isolation, PBMCs or SKILs were stimulated with IDO or PD-L1 peptide, and cytokine-producing T cells were sorted using the IFN-γ or TNF-α Secretion Assay—Cell Enrichment and Detection kit (Miltenyi Biotec).

### Cytokine-production profile of PD-L1- and IDO-specific T cells by intracellular staining

To assess the T cell cytokine-production profile, isolated and expanded IDO- and PD-L1-specific T cell cultures were stimulated for 5 h with peptide at 5 μM in a 96-well plate. One hour after the start of the incubation, anti-CD107a–PE (1:133 dilution, clone H4A3, BD Biosciences, 555801) antibody and BD GolgiPlug (1:1,000 dilution, BD Biosciences) were added. After a 5-h incubation, cells were stained using fluorescently labeled surface marker antibodies: anti-CD4–PerCP (1:5 dilution, clone SK3, 345770), anti-CD8–FITC (1:5 dilution, clone SK1, 345772) and anti-CD3–APC-H7 (1:20 dilution, clone SK7, 560275) (all from BD Biosciences). Dead cells were stained using FVS510 (1:250 dilution, BD Biosciences, 564406), followed by overnight fixation and permeabilization using eBioscience Fixation/Permeabilization buffers (00-5123-43, 00-5223-56) according to the manufacturer’s instructions. Cells were then stained intracellularly in eBioscience permeabilization buffer (00-8333-56) with anti-IFN-γ–APC (1:20 dilution, clone 25723.11, BD Biosciences, 341117) and anti-TNF-α–BV421 (1:100 dilution, clone Mab11, BD Biosciences, 562783) antibodies. Samples were analyzed on the FACSCanto II (BD Biosciences) using BD FACSDiva software version 8.0.2. The gating strategy is shown in Supplementary Fig. 3a. For assessing the reactivity against IDO expressing MonoMac1 cells, the appropriate amount of cancer cells was added to the IDO-specific T cells to obtain an effector:target ratio of 4:1 in the 96-well plate and T cell cytokine production was tested after 5 h of co-culture as described above. For HLA-blocking experiments, HLA-blocking antibodies (2 μg ml^−1^) were added directly into wells 30 min before the addition of peptide. Blocking antibodies used were HLA-DR (1:500 dilution, clone L243, Abcam, ab136320), HLA-DQ (1:500 dilution, clone SPV-L3, Abcam, ab23632) and HLA-DP (1:500 dilution, clone B7/21, Abcam, ab20897).

To assess ex vivo T cell reactivity to IDO and PD-L1 peptides in patient PBMCs, cells were thawed and rested for 1–2 d in medium containing DNase I (1 μg ml^−1^, Sigma-Aldrich, 11284932001). PBMCs were then stimulated with peptide at 5 μM in a 96-well plate for 8 h. An hour after the addition of peptide, anti-CD107a–BV421 (3:50 dilution, clone H4A3, BD Biosciences, 328626) antibody and BD GolgiPlug (1:1,000 dilution, BD Biosciences) were added. Surface and intracellular staining was performed as described above. Antibodies used for surface staining were anti-CD3–PE-CF594 (0.8:30 dilution, clone UCHT1, BD Biosciences, 562280), anti-CD4–BV711 (1:30 dilution, clone SK3, BD Biosciences, 563028) and anti-CD8–Qdot605 (1:150 dilution, clone 3B5, Thermo Fisher, Q10009). Antibodies used for intracellular staining were anti-CD137–PE (1:20 dilution, clone 4B4-1 (RUO), BD Biosciences, 555956), anti-IFN-γ–PE-Cy7 (1.5:20 dilution, clone B27, BD Biosciences, 557643) and anti-TNF-α–APC (1:20 dilution, clone Mab11, BD Biosciences, 554514). Samples were acquired on the NovoCyte Quanteon (ACEA Biosciences) and analyzed using NovoExpress software version 1.4.1. To assess vaccine-specific T cell responses, background values observed in unstimulated PBMC samples were subtracted from values observed in peptide-stimulated conditions. Positive response value threshold was set at a difference of 0.2% from the background values. Based on this response cutoff, only TNF-α, CD107a and CD137 responses were detected in this assay. Statistical analysis comparing baseline with on-treatment or post-treatment cytokine profiles was performed using two-sided Wilcoxon matched-pairs signed-rank test. The gating strategy is shown in Supplementary Fig. 3b.

### siRNA-mediated *PD-L1* and *IDO1* silencing

A stealth siRNA duplex for targeted silencing of *PD-L1* (Invitrogen)^52^, a custom silencer select siRNA for targeted silencing of *IDO1* (Ambion) and the recommended silencer select negative control (Ambion) siRNA for mock transfection were used.

The stealth PD-L1 siRNA duplex consisted of the sense sequence 5′-CCUACUGGCAUU-UGCUGAACGCAUU-3′ and the antisense sequence 5′-AAUGCGUUCAGCAAAUGCCAGUAGG-3′. The three silencer IDO siRNA duplexes used were siRNA1 (sense sequence, 5′-ACAUCUGCCUGAUCUCAUATT-3′; antisense sequence, 5′-UAUGAGAUCAGGCAGAUGUTT-3′), siRNA2 (sense sequence, 5′-CCACGAUCAUGUGAACCCATT-3′; antisense sequence, 5′-UGGGUUCACAUGAUCG-UGGAT-3′) and siRNA3 (sense sequence, 5′-CGAUCAUGUGAACCCAAAATT-3′; antisense sequence, 5′-UUUUGGGUUCACAUGAUCGTG-3′). For *PD-L1* or *IDO1* silencing, cancer cells were electroporated with 0.025 nmol of each siRNA duplex as previously described^53^. For *PD-L1*-silencing experiments, cancer cells were treated with IFN-γ (500 U ml^−1^, PeproTech) 1 h after electroporation. Electroporated cells were used as target cells in ELISPOT and ICS assays 24 h or 48 h after siRNA electroporation.

### RT–qPCR

Total RNA was extracted using the RNeasy Plus Mini kit (Qiagen, 74134) following the manufacturer’s instructions. RNA concentration was quantified using a NanoDrop 2000 (Thermo Fisher Scientific), and a total of 1,000 ng RNA was reverse transcribed with the High-Capacity cDNA Reverse Transcription Kit (Applied Biosystems, 4368814) using 1,000 ng input RNA for transcription. Real-time qPCR analyses were performed using the TaqMan Gene Expression Assay on a Roche LightCycler 480 instrument. RT–qPCR assays were performed with a minimum of three technical replicates and analyzed using the ΔCt method as described in Bookout et al.^54^ with normalization of *IDO1* expression (primer ID, Hs00984148_m1) or *PD-L1* expression (primer ID, Hs001125296_m1) to the expression level of the housekeeping gene *POLR2A* (primer ID, Hs00172187_m1) and the control sample (mock). For low-concentration samples with no amplification, Ct was set to 40. Controls without reverse transcriptase were used as controls for specific amplifications. *P* values were determined using two-tailed parametric *t*-tests.

### *BRAF*-mutational status and PD-L1 status at baseline in all patients

A library of historical FFPE biopsies were assessable for all patients and analyzed locally at Herlev and Gentofte University Hospital by experienced pathologists for *BRAF* status and PD-L1 expression on tumor cells.

The *BRAF* analysis was carried out with real-time PCR using the EntroGen BRAF Mutation Analysis Kit II (BRAFX-RT64, CE-IVD) to specifically detect mutations corresponding to V600D, V600E and V600K in the BRAF protein.

PD-L1 status was assessed using the monoclonal rabbit anti-PD-L1 antibody (clone 28.8, PD-L1 IHC 28-8 pharmDx) in FFPE biopsies following the manufacturer’s instructions. Patients were considered positive for PD-L1 with expression levels ≥1% and negative for PD-L1 with expression levels <1%.

### Human leukocyte antigen type

Blood samples from all 30 patients were genotyped for class I (HLA-A, HLA-B, HLA-C) and three class II types (HLA-DRB1, HLA-DQA1, HLA-DQB1) using LinkSēq HLA Typing Kits (Thermo Fisher, 1580C). These test kits are based on real-time PCR using allele-specific exponential amplification (sequence-specific primers) followed by melting curve analyses.

### Immunohistochemistry simplex: Immunoscore CR

IHC staining was performed using a qualified Ventana Benchmark XT with four different steps: (1) antigen retrieval; (2) staining with the following primary antibodies: anti-CD3 antibody (clone HDX2; provider, HalioDx; HD-FG-000013; 10931065/10636667; concentration, 0.25 µg ml^−1^), anti-CD8 antibody (clone HDX3; provider, HalioDx; HD-FG-000019; 10931069/10337710/10639301; concentration, 0.5 µg ml^−1^), anti-IDO monoclonal antibody (clone VINC3IDO; provider, Thermo Fisher Scientific; 14-9750-82; E25003-101; concentration, 0.05 µg ml^−1^), anti-HLA class 1 ABC antibody (clone, EMR8-5; provider, Abcam; ab70328; GR3248333/GR3186494; concentration, 0.5 µg ml^−1^), anti-HLA-DR/DP/DQ/DX antibody (clone CR3/43; provider, Santa Cruz; sc-53302; L1714; concentration, 1 µg ml^−1^) and anti-PD-L1 antibody (clone HDX3; provider, HalioDx; HD-FG-000035; 106312810/106312816; concentration, 3.3 µg ml^−1^); (3) detection with a secondary antibody using the ultraView Universal DAB Detection kit; and (4) counterstaining using Hematoxylin & Bluing Reagent (staining of cellular nuclei). Control slides were systematically included in each staining run to permit quality control of the obtained measurements. Following coverslipping, slides were scanned with the NanoZoomer-XR to generate digital images (20×).

Two consecutive slides were specifically used to perform Immunoscore CR TL staining of CD3^+^ and CD8^+^ cells.

### Digital pathology of T lymphocytes

The digital pathology for Immunoscore CR TL allowed the quantification of positive cells (in cells per mm^2^) into the core tumor and invasive margin if present. Each sample was analyzed using the HalioDx Digital Pathology Platform.

### Digital pathology and pathologist analysis of IDO

Digital pathology for IDO allowed the quantification of stained area (in mm^2^) into the whole tumor. Each sample was analyzed using the HalioDx Digital Pathology Platform.

Analysis of IDO^+^ cells was performed by a pathologist. Results were expressed as *an H* score from 0 to 300. The score is obtained by the formula 3 × percentage of cells with strong staining + 2 × percentage of cells with moderate staining + percentage of cells with weak staining.

### Digital pathologist analysis of MHC-I and MHC-II

Analysis of MHC-I^+^ and MHC-II^+^ cells was performed by a pathologist. Results are expressed as a percentage of positive tumor cells and a percentage of positive cells in the stroma.

### Digital pathology Immunoscore immune checkpoint (CD8^+^ and PD-L1^+^)

The Immunoscore CR IC test allowed the quantification of CD8^++^ cell density in the whole tumor and a CD8^+^-centered proximity index (which corresponds to the percentage of CD8^++^ cells that have at least one PD-L1^+^ cell in the neighborhood) at different cutoff distances (20 µm, 40 µm, 60 µm and 80 µm).

### Pathologist analysis of PD-L1

A pathologist performed analysis of PD-L1^+^ cells. Positivity of a viable tumor cell was considered when partial or complete cell membrane staining was observed (more than 10% of the tumor cell membrane). Results were expressed as a percentage.

### NanoString RNA profiling and Immunosign

RNA was extracted from FFPE tissues using Qiagen RNeasy FFPE extraction kits (Qiagen). Annotations from the pathologist performing H&E staining were used to guide removal of normal tissue from the slides by macrodissection before nucleic acid extraction, which occurred after tissue deparaffinization and lysis. Each extracted RNA sample was independently quantified using a NanoDrop spectrophotometer (NanoDrop Technologies) and qualified (Agilent Bioanalyzer). Degradation was quantified as the percentage of RNA fragments smaller than 300 bp using the RNA 6000 Nano kit (Agilent Bioanalyzer). Good sample quality was defined as less than 50% of RNA fragments being 50–300 bp in size.

RNA expression profiling was performed using the nCounter PanCancer Immune Profiling Panel from NanoString (NanoString Technologies). The PanCancer Immune Profiling Panel contains 776 probes and is supplemented with six genes to complete HalioDx Immunosign targets.

Hybridization was performed according to the manufacturer’s instructions. Hybridized probes were then purified and immobilized on a streptavidin-coated cartridge using the nCounter Prep Station (NanoString Technologies). Data collection was carried out on the nCounter Digital Analyzer (NanoString Technologies) following the manufacturer’s instructions to count individual fluorescent barcodes and quantify target RNA molecules present in each sample. For each assay, a scan of 490 fields of view was performed.

Raw data from the NanoString nCounter were processed using NanoString recommendations.

Quality control enables to retain data of good quality with a binding density that ranges between 0.05 and 2.25. The linearity of positive controls was checked using the *R*^2^ value of regression between the counts and the concentrations of positive controls. Samples that showed *R*^2^ < 0.75 were flagged and removed from the analysis. The background was removed using a thresholding method at the mean + 2 s.d. of negative controls. Raw counts were normalized using a positive normalization factor. Samples showing positive normalization factors outside the range of 0.3–3 were removed from the analysis. A second normalization was performed using the housekeeping gene normalization factor. Only the most stable housekeeping genes were selected for this normalization step using the variance-versus-mean relationship. All samples showing a normalization factor outside the range of 0.1–10 were removed from the analysis. All statistical analyses were performed on normalized counts using R software (version 2.6.2, 2019-12-12).

### T cell receptor variable β chain sequencing

To track longitudinal immune responses to therapy, genomic DNA was extracted from longitudinal pre- and post-treatment PBMCs (five patients), pre- and post-treatment biopsies (five patients) (both FFPE and treated with RNAlater) and IDO- and PD-L1-specific T cell cultures from PBMCs (five patients) or SKILs (one patient). Three clonal (two IDO- and one PD-L1-) specific cultures were also sequenced to confirm clonal purity of cultures.

DNA from PBMCs or RNAlater-treated biopsies was extracted with the DNeasy Blood and Tissue kit (Qiagen, 69504), DNA from sorted IDO and PD-L1-specific T cells from either PBMCs or SKILs was extracted using the QIAamp DNA Micro kit (Qiagen, 565304), and DNA from PFFE biopsies was extracted using the Maxwell RSC DNA FFPE kit (Promega, AS1450).

Immunosequencing of the CDR3 regions of human TCRβ chains was performed using the immunoSEQÒ Assay (Adaptive Biotechnologies). Extracted genomic DNA was amplified in a bias-controlled multiplex PCR, followed by high-throughput sequencing. Sequences were collapsed and filtered to identify and quantitate the absolute abundance of each unique TCRβ CDR3 region for further analysis as previously described^55–57^.

### Statistical analyses of TCRβ sequencing results

Two quantitative components of diversity were compared across samples in this study. First, Simpson clonality was calculated on productive rearrangements by $\sqrt {\mathop {\sum}\nolimits_{i = 1}^R {p_i^2} }$, where *R* is the total number of rearrangements and _*pi*_ is the productive frequency of rearrangement *i*. Values of Simpson clonality range from 0 to 1 and measure how evenly receptor sequences (rearrangements) are distributed among a set of T cells. Clonality values approaching 0 indicate a very even distribution of frequencies, whereas values approaching 1 indicate an increasingly asymmetric distribution in which a few clones are present at high frequencies.

Second, sample richness was calculated as the number of unique productive rearrangements in a sample after computationally downsampling to a common number of T cells to control for variation in sample depth or T cell fraction. Repertoires were randomly sampled without replacement five times, and we report the mean number of unique rearrangements.

The T cell fraction was calculated by taking the total number of T cell templates and dividing by the total number of nucleated cells. The total number of nucleated cells was derived from reference genes using the immunoSEQ Analyzer version 3.

To identify enriched vaccine clones in each patient, rearrangement frequencies in their baseline PBMCs and each IDO/PD-L1-sorted T cell sample were compared using a binomial distribution framework as previously described^58^. In brief, for each clone, we performed a two-sided test to determine whether frequencies were the same in the patient’s periphery and a PD-L1- or IDO-specific T cell sample. The Benjamini–Hochberg procedure was used to control the false discovery rate at 0.01 (ref. ^59^). Clonal expansion in post-treatment samples was similarly assessed using this differential abundance framework, but an IDO/PD-L1-specific T cell sample was replaced with a post-treatment series sample. In biopsies, the six series frequencies were compared to those of baseline tissue. Lastly, vaccine-associated clones were tracked in each PBMC and tissue sample by summing the frequency of each rearrangement enriched in either PD-L1- or IDO-specific T cells. All statistical analyses were performed in R version 3.6.1.

### Reporting Summary

Further information on research design is available in the Nature Research Reporting Summary linked to this article.

## Online content

Any methods, additional references, Nature Research reporting summaries, source data, extended data, supplementary information, acknowledgements, peer review information; details of author contributions and competing interests; and statements of data and code availability are available at 10.1038/s41591-021-01544-x.

## Supplementary information

Supplementary Information Supplementary Figs. 1–3 and Tables 1–6

Reporting Summary

## Data Availability

This clinical trial was registered at https://www.clinicaltrials.gov/ before patient enrollment (clinical trial identifier NCT03047928). Each vaccine was composed of 100 µg IO102, a 21-amino-acid peptide (DTLLKALLEIASCLEKALQVF) from the IDO peptide, and 100 µg IO103, a 19-amino-acid peptide (FMTYWHLLNAFTVTVPKDL) from the signal peptide of PD-L1. TCR sequencing data are available from Adaptive Biotechnologies. Upon request, the CCIT-DK office will provide a username and a password to access the designated data within approximately 2–4 weeks (https://www.herlevhospital.dk/ccit-denmark/find-us/Sider/Contact-information.aspx). All requests for the remaining data including raw data and analyzed data and materials will, within a reasonable time frame, be reviewed by the CCIT-DK office (https://www.herlevhospital.dk/ccit-denmark/find-us/Sider/Contact-information.aspx) to verify whether the request is subject to any intellectual property or obligations. Patient-related data not included in the paper were generated as part of clinical trials and may be subject to patient confidentiality. Any data and materials that can be shared will be released via a material-transfer agreement. The following database was used in the study: https://research.regionh.dk/da/publications/the-danish-metastatic-melanoma-database-dammed(32749d99-095f-4cae-b5de-769bae27f01e).html.

## References

[1] Robert C (2019). Pembrolizumab versus ipilimumab in advanced melanoma (KEYNOTE-006): post-hoc 5-year results from an open-label, multicentre, randomised, controlled, phase 3 study. Lancet Oncol..

[2] Weber, J. S., Postow, M., Lao, C. D. & Schadendorf, D. Management of adverse events following treatment with anti-programmed death-1 agents. *Oncologist* **21**, 1230–1240 (2016). 10.1634/theoncologist.2016-0055 PMC5061539 27401894

[3] Larkin J (2019). Five-year survival with combined nivolumab and ipilimumab in advanced melanoma. N. Engl. J. Med..

[4] Ascierto PA (2017). Efficacy of BMS-986016, a monoclonal antibody that targets lymphocyte activation gene-3 (LAG-3), in combination with nivolumab in pts with melanoma who progressed during prior anti-PD-1/PD-L1 therapy (mel prior IO) in all-comer and biomarker-enriched populations. Ann. Oncol..

[5] Angeles L (2018). Warming ‘cold’ melanoma with TLR9 agonists. Cancer Discov..

[6] Mullinax JE (2018). Combination of ipilimumab and adoptive cell therapy with tumor-infiltrating lymphocytes for patients with metastatic melanoma. Front. Oncol..

[7] Ott PA (2020). A phase Ib trial of personalized neoantigen therapy plus anti-PD-1 in patients with advanced melanoma, non-small cell lung cancer, or bladder cancer. Cell.

[8] Ott PA (2017). An immunogenic personal neoantigen vaccine for patients with melanoma. Nature.

[9] Andersen MH (2019). Anti-cancer immunotherapy: breakthroughs and future strategies. Semin. Immunopathol..

[10] Munir S, Andersen GH, Svane IM, Andersen MH (2013). The immune checkpoint regulator PD-L1 is a specific target for naturally occurring CD4^+^ T cells. Oncoimmunology.

[11] Ahmad SM, Borch TH, Hansen M, Andersen MH (2016). PD-L1-specific T cells. Cancer Immunol. Immunother..

[12] Andersen MH (2012). The specific targeting of immune regulation: T-cell responses against indoleamine 2,3-dioxygenase. Cancer Immunol. Immunother..

[13] Sørensen RB (2011). Spontaneous cytotoxic T-cell reactivity against indoleamine 2,3-dioxygenase-2. Cancer Res..

[14] Ahmad SM, Larsen SK, Svane IM, Andersen MH (2014). Harnessing PD-L1-specific cytotoxic T cells for anti-leukemia immunotherapy to defeat mechanisms of immune escape mediated by the PD-1 pathway. Leukemia.

[15] Andersen MH (2012). CD4 responses against IDO. Oncoimmunology.

[16] Dey S (2020). Peptide vaccination directed against IDO1-expressing immune cells elicits CD8^+^ and CD4^+^ T-cell-mediated antitumor immunity and enhanced anti-PD1 responses. J. Immunother. Cancer.

[17] Ellebaek, E. et al. The Danish metastatic melanoma database (DAMMED): a nation-wide platform for quality assurance and research in real-world data on medical therapy in Danish melanoma patients. *Cancer Epidemiol*. **73**, 101943 (2021). 10.1016/j.canep.2021.101943 33962356

[18] Larkin J (2015). Combined nivolumab and ipilimumab or monotherapy in untreated melanoma. N. Engl. J. Med..

[19] Godard B (2004). Optimization of an Elispot assay to detect cytomegalovirus-specific CD8^+^ T lymphocytes. Hum. Immunol..

[20] Meier A (2005). Spontaneous T-cell responses against peptides derived from the Taxol resistance-associated gene-3 (TRAG-3) protein in cancer patients. Cancer Immunol. Immunother..

[21] Calarota SA (2008). HIV-1-specific T cell precursors with high proliferative capacity correlate with low viremia and high CD4 counts in untreated individuals. J. Immunol..

[22] Wolchok JD (2017). Overall survival with combined nivolumab and ipilimumab in advanced melanoma. N. Engl. J. Med..

[23] Robert C (2015). Nivolumab in previously untreated melanoma without *BRAF* mutation. N. Engl. J. Med..

[24] Iii CHK (2019). Age correlates with response to anti-PD1, reflecting age-related differences in intratumoral effector and regulatory T-cell populations. Clin. Cancer Res..

[25] Bastholt L (2019). Age favoured overall survival in a large population-based Danish patient cohort treated with anti-PD1 immune checkpoint inhibitor for metastatic melanoma. Eur. J. Cancer.

[26] Yan, X., Tian, X., Wu, Z. & Han, W. Impact of age on the efficacy of immune checkpoint inhibitor-based combination therapy for non-small-cell lung cancer: a systematic review and meta-analysis. *Front. Oncol*. **10**, 1671 (2020). 10.3389/fonc.2020.01671 PMC7538697 33072551

[27] Huang XZ (2020). Efficacy of immune checkpoint inhibitors and age in cancer patients. Immunotherapy.

[28] Khozin S, Blumenthal GM, Pazdur R (2017). Real-world data for clinical evidence generation in oncology. J. Natl Cancer Inst..

[29] Ribas A (2017). Oncolytic virotherapy promotes intratumoral T cell infiltration and improves anti-PD-1 immunotherapy. Cell.

[30] Long G (2020). 429 Long-term analysis of MASTERKEY-265 phase 1b trial of talimogene laherparepvec (T-VEC) plus pembrolizumab in patients with unresectable stage IIIB–IVM1c melanoma. J. Immunother. Cancer.

[31] Long GV (2019). Epacadostat plus pembrolizumab versus placebo plus pembrolizumab in patients with unresectable or metastatic melanoma (ECHO-301/KEYNOTE-252): a phase 3, randomised, double-blind study. Lancet Oncol..

[32] Iversen TZ (2014). Long-lasting disease stabilization in the absence of toxicity in metastatic lung cancer patients vaccinated with an epitope derived from indoleamine 2,3 dioxygenase. Clin. Cancer Res..

[33] Kjeldsen JW (2018). Durable clinical responses and long-term follow-up of stage III–IV non-small-cell lung cancer (NSCLC) patients treated with IDO peptide vaccine in a phase I study—a brief research report. Front. Immunol..

[34] Sahin U (2020). An RNA vaccine drives immunity in checkpoint-inhibitor-treated melanoma. Nature.

[35] Sørensen RB (2011). Indoleamine 2,3-dioxygenase specific, cytotoxic T cells as immune regulators. Blood.

[36] Søorensen RB (2009). The immune system strikes back: cellular immune responses against indoleamine 2,3-dioxygenase. PLoS ONE.

[37] Munir S (2012). Natural CD4^+^ T-cell responses against indoleamine 2,3-dioxygenase. PLoS ONE.

[38] Munir S (2013). HLA-restricted CTL that are specific for the immune checkpoint ligand PD-L1 occur with high frequency in cancer patients. Cancer Res..

[39] Andersen MH (2019). The targeting of tumor-associated macrophages by vaccination. Cell Stress.

[40] Andersen R (2016). Long-lasting complete responses in patients with metastatic melanoma after adoptive cell therapy with tumor-infiltrating lymphocytes and an attenuated IL2 regimen. Clin. Cancer Res..

[41] Jørgensen NG (2020). Peptide vaccination against PD-L1 with IO103 a novel immune modulatory vaccine in multiple myeloma: a phase I first-in-human trial. Front. Immunol..

[42] Tumeh PC (2014). PD-1 blockade induces responses by inhibiting adaptive immune resistance. Nature.

[43] Wang T (2018). A cancer vaccine-mediated postoperative immunotherapy for recurrent and metastatic tumors. Nat. Commun..

[44] Vilain RE (2017). Dynamic changes in PD-L1 expression and immune infiltrates early during treatment predict response to PD-1 blockade in melanoma. Clin. Cancer Res..

[45] Andersen MH (2018). The balance players of the adaptive immune system. Cancer Res..

[46] Diskin B (2020). PD-L1 engagement on T cells promotes self-tolerance and suppression of neighboring macrophages and effector T cells in cancer. Nat. Immunol..

[47] Ahmad SM, Svane IM, Andersen MH (2014). The stimulation of PD-L1-specific cytotoxic T lymphocytes can both directly and indirectly enhance antileukemic immunity. Blood Cancer J..

[48] Munir Ahmad S (2016). PD-L1 peptide co-stimulation increases immunogenicity of a dendritic cell-based cancer vaccine. Oncoimmunology.

[49] Pardoll DM (2012). The blockade of immune checkpoints in cancer immunotherapy. Nat. Rev. Cancer.

[50] Moodie Z (2010). Response definition criteria for ELISPOT assays revisited. Cancer Immunol. Immunother..

[51] Donia M (2011). Characterization and comparison of ‘standard’ and ‘young’ tumor infiltrating lymphocytes for adoptive cell therapy at a Danish translational research institution. Scand. J. Immunol..

[52] Hobo W (2010). siRNA silencing of PD-L1 and PD-L2 on dendritic cells augments expansion and function of minor histocompatibility antigen-specific CD8^+^ T cells. Blood.

[53] Met Ö, Balslev E, Flyger H, Svane IM (2011). High immunogenic potential of p53 mRNA-transfected dendritic cells in patients with primary breast cancer. Breast Cancer Res. Treat..

[54] Bookout AL, Cummins CL, Mangelsdorf DJ, Pesola JM, Kramer MF (2006). High‐throughput real‐time quantitative reverse transcription PCR. Curr. Protoc. Mol. Biol..

[55] Robins HS (2009). Comprehensive assessment of T-cell receptor β-chain diversity in αβ T cells. Blood.

[56] Carlson CS (2013). Using synthetic templates to design an unbiased multiplex PCR assay. Nat. Commun..

[57] Robins H (2012). Ultra-sensitive detection of rare T cell clones. J. Immunol. Methods.

[58] DeWitt WS (2015). Dynamics of the cytotoxic T cell response to a model of acute viral infection. J. Virol..

[59] Benjamini Y, Gavrilov Y (2009). A simple forward selection procedure based on false discovery rate control. Ann. Appl. Stat..

