# Author Correction: A phase 1/2 trial of an immune-modulatory vaccine against IDO/PD-L1 in combination with nivolumab in metastatic melanoma

**DOI:** 10.1038/s41591-022-01771-w

**Published:** 2022-03-08

**Authors:** Julie Westerlin Kjeldsen, Cathrine Lund Lorentzen, Evelina Martinenaite, Eva Ellebaek, Marco Donia, Rikke Boedker Holmstroem, Tobias Wirenfeldt Klausen, Cecilie Oelvang Madsen, Shamaila Munir Ahmed, Stine Emilie Weis-Banke, Morten Orebo Holmström, Helle Westergren Hendel, Eva Ehrnrooth, Mai-Britt Zocca, Ayako Wakatsuki Pedersen, Mads Hald Andersen, Inge Marie Svane

**Affiliations:** 1 National Center for Cancer Immune Therapy (CCIT-DK), Department of Oncology, Copenhagen University Hospital, Herlev, Denmark; 2 IO Biotech Aps, Copenhagen, Denmark; 3 Department of Clinical Physiology and Nuclear Medicine, Copenhagen University Hospital, Herlev, Denmark; 4 Department of Immunology and Microbiology, University of Copenhagen, Copenhagen, Denmark

**Keywords:** Phase II trials, Melanoma, Translational research, Peptide vaccines

Correction to: *Nature Medicine* 10.1038/s41591-021-01544-x, published online 9 December 2021.

This paper was originally published under standard Springer Nature license (© The Author(s), under exclusive licence to Springer Nature America, Inc.). It is now available as an Open Access paper under a Creative Commons Attribution 4.0 International license, © The Author(s). The error has been corrected in the HTML and PDF versions of the article.

