# Supplementary material for: A phase 1/2 trial of an immune-modulatory vaccine against IDO/PD-L1 in combination with nivolumab in metastatic melanoma
Source: Nat Med. 2021 Dec 9;27(12):2212–23. doi: 10.1038/s41591-021-01544-x (PMC8904254; doi:10.1038/s41591-021-01544-x)
Supplement: Supplementary file 1 — Supplementary Figs. 1–3 and Tables 1–6 [file 41591_2021_1544_MOESM1_ESM.pdf]

nature portfolio

https://doi.org/10.1038/s41591-021-01544-x

Supplementary information

A phase 1/2 trial of an immune-modulatory vaccine against IDO/PD-L1 in combination with nivolumab in metastatic melanoma

In the format provided by the
authors and unedited

Supplementary figure 1

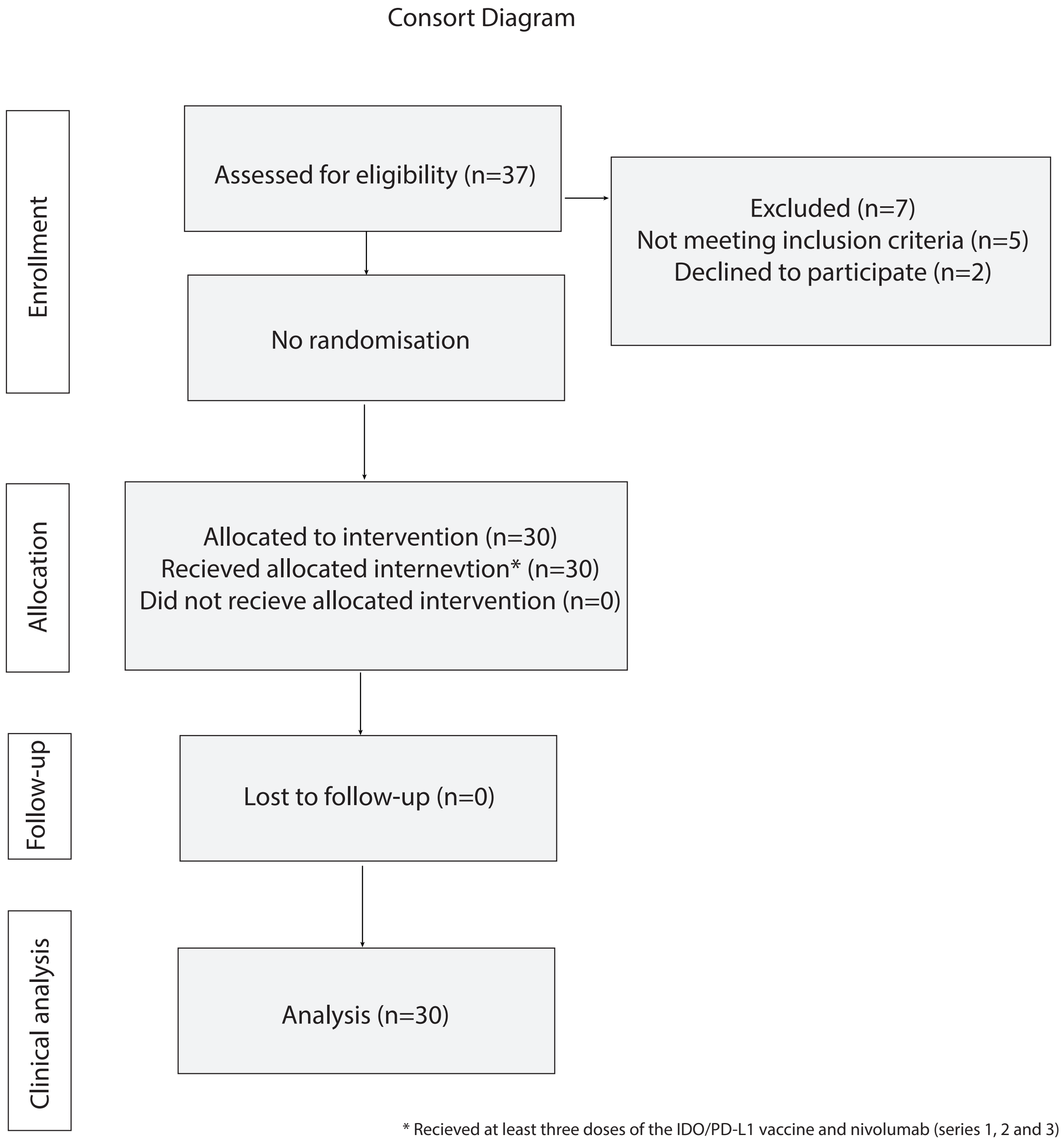

Supplementary figure 1. Consort diagram. A total of 37 patients with metastatic melanoma were consented to the study. A total of 30 patients were enrolled and all 30 patients received allocated study treatment.

Supplementary figure 2

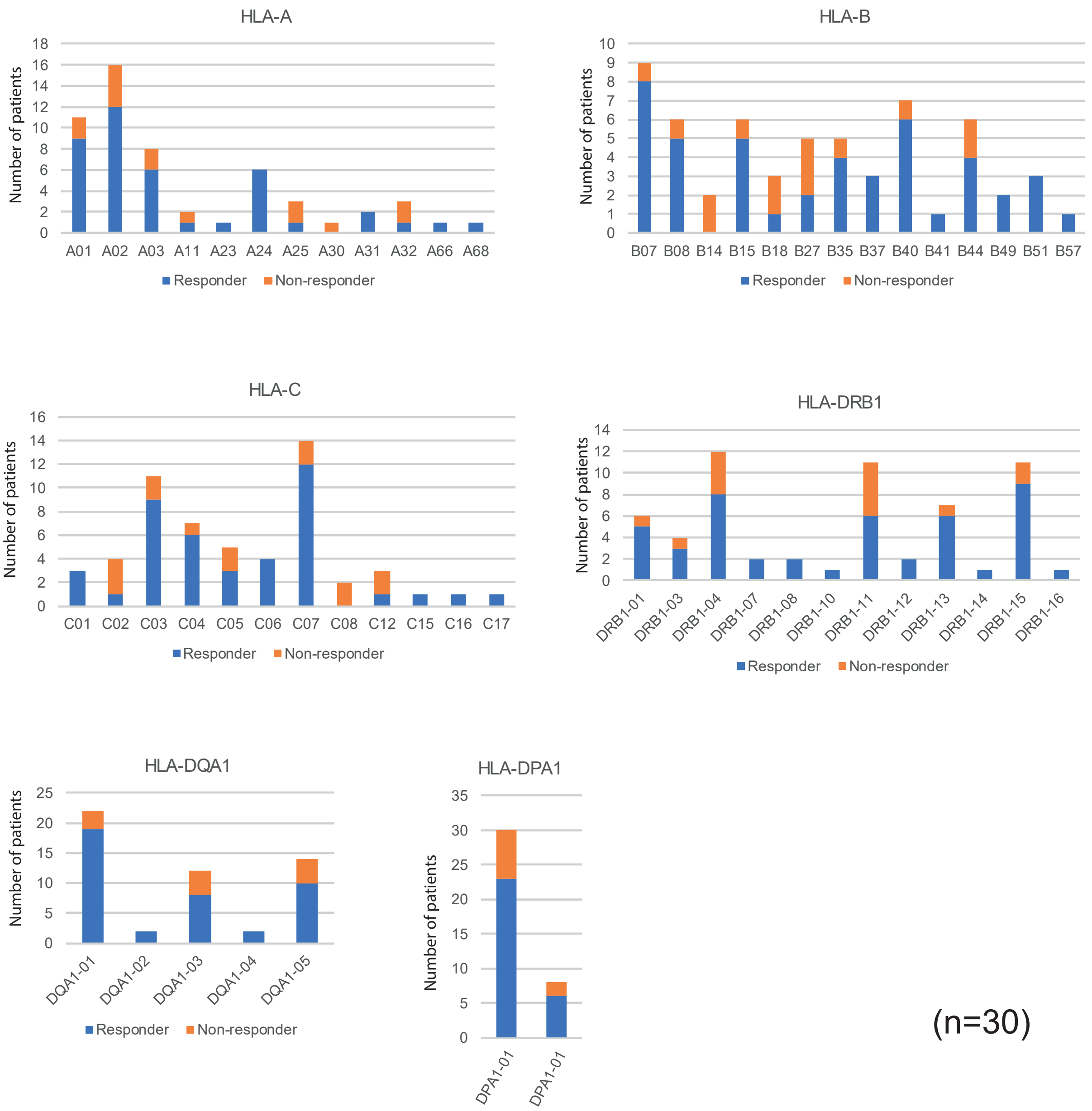

Supplementary figure 2. HLA genotypes and clinical response. Bar plots indicating HLA-genotype and clinical response. Blue bars represent responders (CR+PR) and orange bars represent non-responders (PD) (n=30).

Supplementary figure 3

a

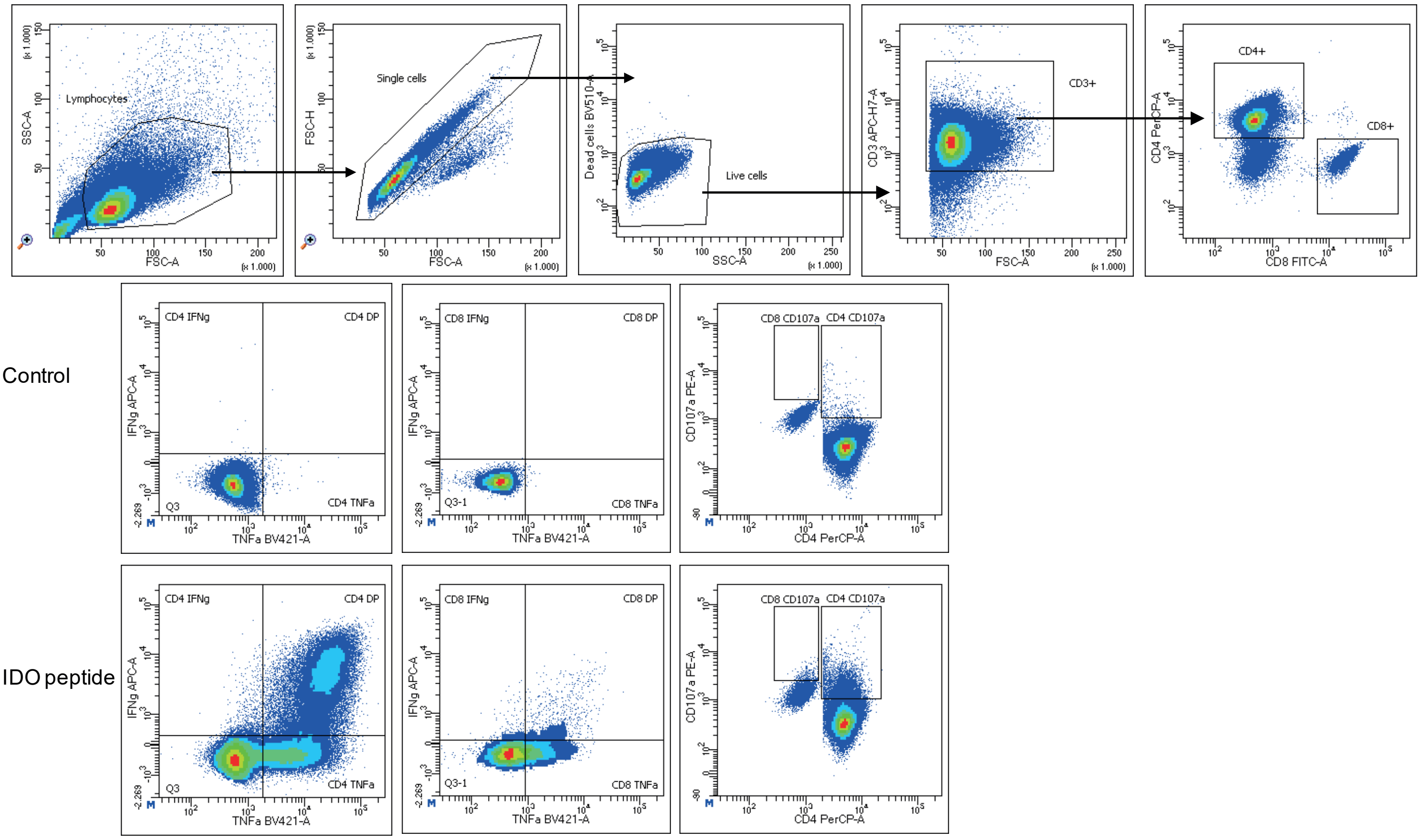

b

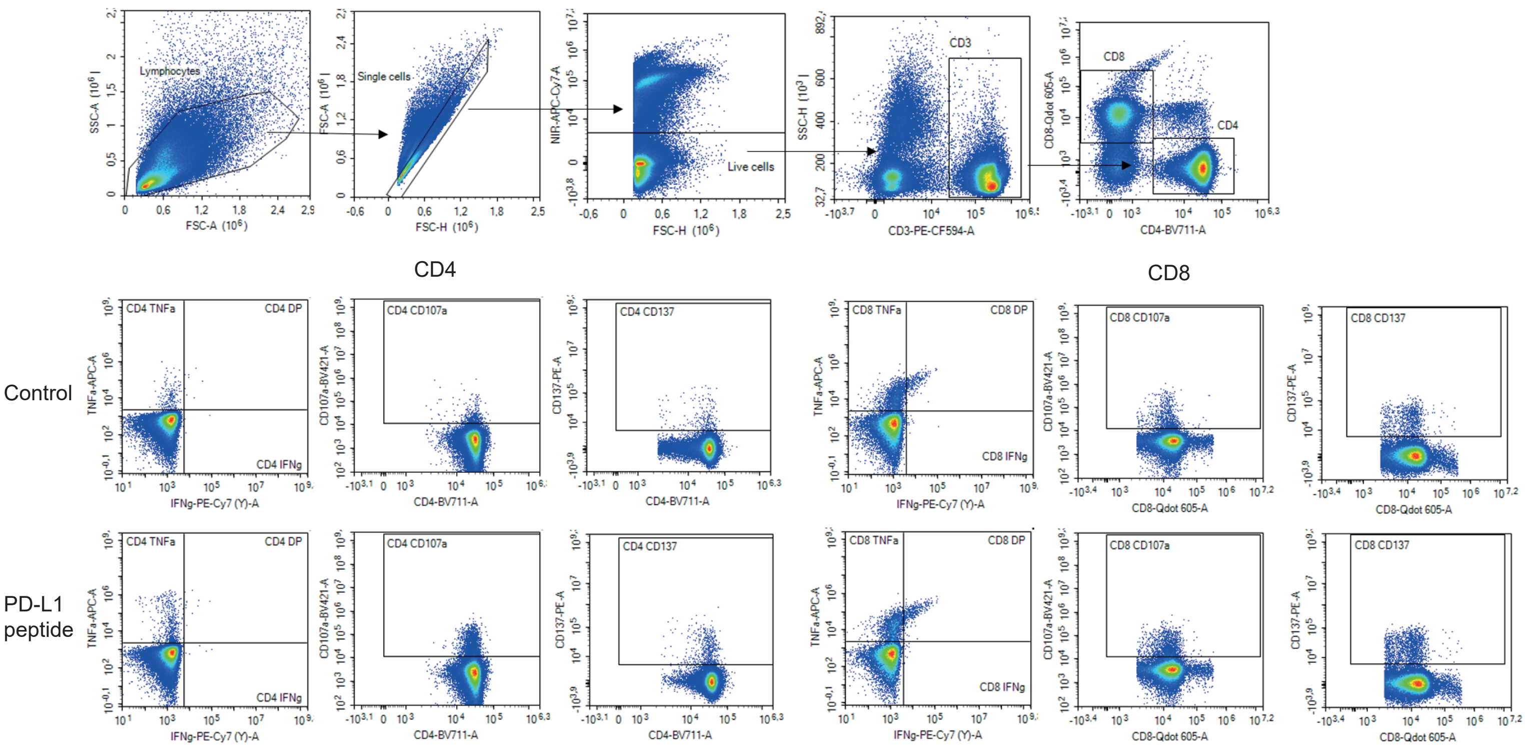

Supplementary figure 3. Gating strategy. a) Gating strategy used in cytokine production profile of IDO and PD-L1 specific T cells by intracellular staining. **b)** Gating strategy used for data presented in Extended Figure 8 for assessment of IDO and PD-L1 specific T cells by intracellular staining of PBMCs ex vivo.

Supplementary table 1. Doses of vaccines and nivolumab administered and reason to stop treatment plus subsequent therapy (n=30)

Patient ID	BOR[1]	Number vaccines series	Number nivolumab series (3mg/kg)	Number nivolumab series (6 mg/kg)	Reason stop treatment	Subsequent therapy
MM1636.01	CR	15	24	5	Grade 3 rash	
MM1636.02	PD	5	5		Progression	Dabrafenib/Trametinib
MM1636.03	CR	15	24	9	Maximum benefit	
MM1636.04	PD	5	5		Progression	Radiotherapy, Temodal
MM1636.05	CR	13	21		Grade 2 pneumonitis	
MM1636.06	CR	15	24	9	Progression	Dabrafenib/Trametinib
MM1636.07	CR	8	12	2	Grade 2 pneumonitis	Reinduction Nivolumab
MM1636.08	PR	11	16		Progression	Ipilimumab, Radiotherapy
MM1636.09	PD	5	5		Progression	
MM1636.10	PR	15	24	7	Progression	Encorafenib/Binimetinib
MM1636.11	PD	3	3		Progression	Dabrafenib/Trametinib, Ipilimumab
MM1636.12	CR	15	24	8	Maximum benefit	
MM1636.13	PR	15	24	13	Reached 2 years of treatment	
MM1636.14	CR	11	16		Grade 2 colitis	
MM1636.15	PR	15	24	11	Reached 2 years of treatment	
MM1636.16	PR	9	12		Progression	T cell therapy CT ID: NCT02278887
MM1636.17	PD	6	6		Progression	
MM1636.18	CR	5	5		Grade 5 toxicity due to nivolumab	
MM1636.20	CR	11	18	2	Maximum benefit	
MM1636.22	CR	15	24	1	Grade 3 arthralgia	
MM1636.23	CR	15	24	3	Maximum benefit	
MM1636.24	CR	15	24		Maximum benefit	
MM1636.27	PR	15	24	2	Treatment ongoing	
MM1636.29	PR	15	24	2	Treatment ongoing	
MM1636.34	PD	3	3		Progression	
MM1636.35	PR	21	13		Treatment ongoing	
MM1636.37	PR	7	8		Progression	Encorafenib/Binimetinib
MM1636.38	CR	18	12		Treatment ongoing	
MM1636.39	PR	16	11		Treatment ongoing	
MM1636.42	PR	7	6		Treatment ongoing	

[1]BOR = Best Overall Response

Supplementary table 2. Patient baseline characteristics for all patients (n=30)

Patient ID	Gender	Age	Previous treatment	Metastatic sites at baseline	Sum of target lesions at baseline (mm)	M-stage AJCC 7th edition	M-stage AJCC 8th edition	PD-L1 status (clone 28.8)	BRAF status	LDH at baseline (U/L)
MM1636.01	M	85	None	LN, SC, adrenal glands, peritoneal carcinomatosis, bone	33	M1c	M1c(1)	<1%	Wildtype	279
MM1636.02	F	69	None	LN, tumor along iliaca artery, SC	216	M1c	M1c(1)	<1%	Mutated	1510
MM1636.03	F	79	None	Pericardial fat, Lung	60	M1c	M1c(1)	>1%	Mutated	260
MM1636.04	F	77	1. line Ipilimumab	LN, Lung	62	M1b	M1b(0)	<1%	Wildtype	183
MM1636.05	M	72	None	LN, peritoneal carcinomatosis, pleural carcinomatosis, SC	88	M1c	M1c(1)	>1%	Wildtype	324
MM1636.06	F	67	Adjuvant Ipilimumab	LN	19	M1a	M1a(0)	>1%	Mutated	180
MM1636.07	M	72	None	LN	43	M1a	M1a(0)	>1%	Wildtype	134
MM1636.08	F	76	1. line Ipilimumab	LN, SC, tumor near liver, tumor in pelvis, bone marrow tibia	191	M1c	M1c(1)	<1%	Wildtype	269
MM1636.09	F	72	none	Lung, liver, adrenal gland	72	M1c	M1c(0)	<1%	Wildtype	255
MM1636.10	M	46	none	Lung, pleura, adrenal gland	11	M1c	M1c(0)	>1%	Mutated	132
MM1636.11	F	67	none	LN, SC	21	M1a	M1a(0)	<1%	Mutated	187
MM1636.12	F	77	none	LN	26	M1a	M1a(0)	>1%	Mutated	251
MM1636.13	F	59	none	LN, SC, Lung, liver, bone	77	M1c	M1c(1)	>1%	Wildtype	252
MM1636.14	M	54	none	LN, SC, gall bladder, lung	76	M1c	M1c(0)	<1%	Mutated	192
MM1636.15	M	80	none	LN, liver, rectal	26	M1c	M1c(1)	<1%	Wildtype	309
MM1636.16	M	46	none	Lung, liver	14	M1c	M1c(0)	> 1%	Mutated	203
MM1636.17	M	77	none	Lung, SC	12	M1b	M1b(0)	> 1%	Wildtype	190
MM1636.18	F	80	none	Lung, SC	23	M1b	M1b(0)	< 1%	Wildtype	164
MM1636.20	M	56	none	LN, liver	44	M1c	M1c(0)	< 1%	Mutated	145
MM1636.22	M	76	none	LN, Lung	73	M1b	M1b(0)	>1%	Wildtype	178
MM1636.23	F	76	none	LN, pleura, spleen	47	M1c	M1c(0)	<1%	Wildtype	173
MM1636.24	F	67	none	adrenal gland	31	M1c	M1c(0)	>1%	Wildtype	168
MM1636.27	M	82	none	SC, intramuscular, liver, LN	55	M1c	M1c(0)	>1%	Mutated	236
MM1636.29	M	74	none	LN, Lung, SC	13	M1b	M1b(0)	> 1%	Wildtype	165
MM1636.34	M	75	none	LN, Lung, liver	41	M1c	M1c(1)	< 1%	Wildtype	314
MM1636.35	M	52	none	lung, liver, SC	15	M1c	M1c(1)	> 1%	Wildtype	206
MM1636.37	F	76	none	LN	42	M1a	M1a(0)	<1%	Mutated	232
MM1636.38	F	53	none	SC	25	M1a	M1a(1)	>1%	Wildtype	270
MM1636.39	M	80	none	lung	34	M1b	M1b(0)	>1%	Wildtype	180
MM1636.42	M	81	none	liver, SC	144	M1c	M1c(1)	>1%	Wildtype	317

**Supplementary table 3. Investigator vs independent central response review (n=30)**			
**Patient ID**	**Investigator review**	**Independent central review**	**Explanation of inconsistencies between RECIST 1.1 response evaluation**
MM1636.01	CR	CR	
MM1636.02	PD	PD	
MM1636.03	CR	CR	
MM1636.04	PD	PD	
MM1636.05	CR	CR	
MM1636.06	CR	CR	
MM1636.07	CR	PR	Baseline PET/CT with two mediastinal lymph nodes as target lesions and no non-target lesions. Investigator asses CR due to normalization of both glands and no FDG uptake. Independent reviewer asses PR due to measurable lymph nodes of respectively 11 mm and 11 mm on same scan.
MM1636.08	PR	PR	
MM1636.09	PD	PD	
MM1636.10	PR	CR	Baseline PET/CT with one target lesion in the lung (16 mm) and non-target lung lesions. Investigator asses PR due to still visible non-target lesions but the target lesion is gone. Independent central reviewer asses CR due to target lesion 0 mm and considers non-target lesions to be non-malignant.
MM1636.11	PD	PD	
MM1636.12	CR	CR	
MM1636.13	PR	CR	Baseline PET/CT with three target lesions: two mediastinal lymph nodes (41 mm and 26 mm) and a liver metastasis (9 mm) and non-target lesions (lymph nodes). All non-target lesions are gone, and the two lymph node target lesions have normalized and the liver metastases is 3 mm. Investigator asses PR due to FDG uptake in one of the mediastinal lymph nodes and it keeps decreasing in size. Independent reviewer asses as CR due to CT normalization of lymph nodes and asses liver metastases of 3 mm to be non-malignant with no FDG uptake.

MM1636.14	CR	CR	
MM1636.15	PR	CR	Baseline PET/CT with two liver target lesions (11 mm and 12 mm) and non-target lesions consisting of other liver metastases and lymph nodes in the mesorectal fat. All target lesions are 0 and all non-target liver metastases are gone. Investigator asses PR due to continued FDG uptake in a normalized mesorectal lymph node. Independent central review asses CR due to CT normalization of non-target lymph nodes and no liver metastases.
MM1636.16	PR	SD	Investigator measures baseline PET/CT target lesion in liver to be 14 mm while independent central review measures it to be 12 mm. No non-target lesions. Investigator review measures lesion to 9 mm while independent central review measure it to 11 mm and therefore asses PR vs SD.
MM1636.17	PD	PD	
MM1636.18	CR	CR	
MM1636.20	CR	CR	
MM1636.22	CR	CR	
MM1636.23	CR	CR	
MM1636.24	CR	CR	
MM1636.27	PR	PR	
MM1636.29	PR	CR	Baseline PET/CT with one target lesion in right lung (13 mm) and no non-target lesions. At baseline cutaneous metastases on left crus, biopsy verified but not detectable on PET/CT scan. Investigator asses best decrease in target a to be 10 mm and a biopsy from cutaneous metastases on crus is without malignancy. Is therefore assessed to be PR. Independent reviewer asses target lesion to decrease to 0 mm and therefore asses patient to be in CR.
MM1636.34	PD	PD	
MM1636.35	PR	PR	
MM1636.37	PR	PR	
MM1636.38	CR	CR	
MM1636.39	PR	PR	
MM1636.42	PR	PR	

**Supplementary table 4. Treatment related adverse events (n=30)**				
	Grade 1-2		Grade 3-4	
**Skin**				
Rash	14	*47%*	1	*3%*
Dry skin	6	*20%*		
Pruritus	6	*20%*		
Vitiligo	4	*13%*		
Vasculitus		*3%*		
**Gastrointestinal**				
Diarrhoea	7	*23%*	*1*	*3%*
Constipation	1	*3%*		
Abdominal pain	1	*3%*		
Colitis	1	*3%*	*1*	*3%*
**Respiratory**				
Pneumonitis	3	*10%*		
Pleural effusion	1	*3%*		
Dyspnea	4	*13%*		
**Endocrinic**				
Adrenal insufficiency	2	*7%*	*1*	*3%*
Hypophysitis	2	*7%*		
hypothyroidisme	1	*3%*		
Hyperthyrpodisme	2	*7%*		
**Musculosceletal**				
Arthralgia	9	*30%*	*2*	*7%*
Myalgia	5	*17%*		
**Laboratory tests**				
Increased amylase	6	*20%*		
Increase ALAT	3	*10%*		
Hyponatremia			*1*	*3%*
**Infusion reaction**	5	*17%*		
**Other**				
Nausea	7	*23%*		
Pruritus	6	*20%*		
Xerostomia	5	*17%*		
Stomatitis	2	*7%*		
Nasal congestion	2	*7%*		
Periorbital oedema	1	*3%*		
Dry eyes	1	*3%*		
Peripheral neuropathy	1	*3%*		
Hearing imparied	1	*3%*		
**Vaccine related**				
Injection site reaction	23	*77%*		
Granuloma at injection site	19	*63%*		
Redness at injection site	6	*20%*		
Pain at injection site	4	*13%*		
Pruritus at injection site	4	*13%*		
Myalgia at injection site	1	*3%*		

Supplementary table 5. HLA type (n=30)

Patient ID	HLA-A	HLA-B	HLA-C	HLA-DRB1	HLA-DQA1	HLA-DPA1	HLA-DQB1
**MM1636.01**	A01 A25	B08 B18	C07 C12	03 15	01 05	01 01	02 06
**MM1636.02**	A01 A03	B27 B44	C02 C05	04 11	03 05	01 01	03 03
**MM1636.03**	A01 A02	B08 B40	C03 C07	03 13	01 05	01 02	02 06
**MM1636.04**	A03 A25	B07 B18	C07 C12	04 15	01 03	01 02	03 06
**MM1636.05**	A01 A03	B08 B49	C07 C07	03 13	01 05	02 02	02 06
**MM1636.06**	A02 A68	B15 B37	C03 C06	01 13	01 01	01 02	05 06
**MM1636.07**	A23 A31	B07 B44	C04 C07	11 15	01 05	01 01	03 06
**MM1636.08**	A02 A26	B35 B51	C04 C05	11 13	01 05	01 01	03 06
**MM1636.09**	A02 A32	B15 B44	C03 C05	04 11	03 05	01 01	03 03
**MM1636.10**	A03 A24	B07 B51	C07 C15	15 16	01 01	01 02	05 06
**MM1636.11**	A02 A25	B18 B27	C02 C12	04 15	01 03	01 01	03 06
**MM1636.12**	A03 A66	B15 B37	C03 C06	04 10	01 03	01 01	03 05
**MM1636.13**	A01 A02	B08 B40	C03 C07	04 13	01 03	01 01	03 06
**MM1636.14**	A02 A03	B07 B51	C01 C07	01 11	01 05	01 01	03 05
**MM1636.15**	A24 A32	B40 B44	C03 C05	04 11	03 05	01 01	03 03
**MM1636.16**	A03 A31	B07 B27	C02 C07	04 12	03 05	01 01	03 03
**MM1636.17**	A02 A30	B14 B27	C02 C08	01 11	01 05	01 01	03 05
**MM1636.18**	A02 A02	B27 B40	C01 C03	01 13	01 01	01 01	05 06
**MM1636.20**	A24 A24	B35 B44	C04 C16	07 08	02 04	01 02	02 04
**MM1636.22**	A01 A02	B07 B49	C07 C07	08 15	01 04	01 01	04 06
**MM1636.23**	A03 A03	B07 B07	C07 C07	01 15	01 01	01 01	05 06
**MM1636.24**	A02 A02	B15 B44	C04 C05	01 04	01 03	01 01	03 05
**MM1636.27**	A02 A02	B40 B41	C03 C17	04 15	01 03	01 01	03 06
**MM1636.29**	A01 A11	B07 B08	C07 C07	04 04	03 03	01 01	03 03
**MM1636.34**	A01 A11	B08 B35	C04 C07	03 11	05 05	01 02	02 03
**MM1636.35**	A02 A02	B15 B40	C01 C03	11 12	05 05	01 01	03 03
**MM1636.37**	A01 A24	B35 B37	C04 C06	11 15	01 05	01 02	03 06
**MM1636.38**	A01 A24	B35 B57	C04 C06	07 14	01 02	01 02	02 05
**MM1636.39**	A02 A24	B15 B15	C03 C03	04 15	01 03	01 01	03 06
**MM1636.42**	A01 A02	B07 B07	C07 C07	15 15	01 01	01 01	06 06

Supplementary table 6. Overview of DTH injections and Skin infiltrating T cells grown				
Patient ID	DTH injections	SKILS grown from mix	SKILS grown from PD-L1 alone	SKILS grown from IDO alone
MM1636.01	mix of PD-L1/IDO * 3	yes	NA	NA
MM1636.03	mix of PD-L1/IDO * 3	yes	NA	NA
MM1636.04	mix of PD-L1/IDO * 3	yes	NA	NA
MM1636.05	mix of PD-L1/IDO * 3	no	NA	NA
MM1636.06	mix of PD-L1/IDO * 3	yes	NA	NA
MM1636.08	PD-L1 alone, IDO alone and mix of PD-L1/IDO	no	NA	NA
MM1636.10	PD-L1 alone, IDO alone and mix of PD-L1/IDO	yes	yes	yes
MM1636.14	PD-L1 alone, IDO alone and mix of PD-L1/IDO	yes	yes	no
MM1636.15	PD-L1 alone, IDO alone and mix of PD-L1/IDO	yes	yes	yes
MM1636.16	PD-L1 alone, IDO alone and mix of PD-L1/IDO	yes	yes	no
MM1636.20	PD-L1 alone, IDO alone and mix of PD-L1/IDO	yes	yes	no
MM1636.22	PD-L1 alone, IDO alone and mix of PD-L1/IDO	yes	yes	no
MM1636.23	PD-L1 alone, IDO alone and mix of PD-L1/IDO	yes	yes	no
MM1636.24	PD-L1 alone, IDO alone and mix of PD-L1/IDO	yes	yes	no
MM1636.27	PD-L1 alone, IDO alone and mix of PD-L1/IDO	yes	no	yes
