# Supplementary material for: A phase 1/2 trial of an immune-modulatory vaccine against IDO/PD-L1 in combination with nivolumab in metastatic melanoma
Source: Nat Med. 2021 Dec 9;27(12):2212–23. doi: 10.1038/s41591-021-01544-x (PMC8904254; doi:10.1038/s41591-021-01544-x)
Supplement: Supplementary file 2 — Reporting Summary [file 41591_2021_1544_MOESM2_ESM.pdf]

nature research

Corresponding author(s): Inge Marie Svane

Last updated by author(s): Sep 16, 2021

Reporting Summary

Nature Research wishes to improve the reproducibility of the work that we publish. This form provides structure for consistency and transparency in reporting. For further information on Nature Research policies, see our Editorial Policies and the Editorial Policy Checklist.

Statistics

For all statistical analyses, confirm that the following items are present in the figure legend, table legend, main text, or Methods section.

n/a	Confirmed	
☐	☒	The exact sample size (n) for each experimental group/condition, given as a discrete number and unit of measurement
☐	☒	A statement on whether measurements were taken from distinct samples or whether the same sample was measured repeatedly
☐	☒	The statistical test(s) used AND whether they are one- or two-sided<br>*Only common tests should be described solely by name; describe more complex techniques in the Methods section.*
☐	☒	A description of all covariates tested
☐	☒	A description of any assumptions or corrections, such as tests of normality and adjustment for multiple comparisons
☐	☒	A full description of the statistical parameters including central tendency (e.g. means) or other basic estimates (e.g. regression coefficient) AND variation (e.g. standard deviation) or associated estimates of uncertainty (e.g. confidence intervals)
☐	☒	For null hypothesis testing, the test statistic (e.g. F, t, r) with confidence intervals, effect sizes, degrees of freedom and P value noted<br>*Give P values as exact values whenever suitable.*
☒	☐	For Bayesian analysis, information on the choice of priors and Markov chain Monte Carlo settings
☒	☐	For hierarchical and complex designs, identification of the appropriate level for tests and full reporting of outcomes
☒	☐	Estimates of effect sizes (e.g. Cohen's d, Pearson's r), indicating how they were calculated

Our web collection on statistics for biologists contains articles on many of the points above.

Software and code

Policy information about availability of computer code

Data collection	Data was collected with:<br>OpenClinica (eCRF) v1.0<br>Microsoft Excel version 2002 on a secure server<br>BD FACSDIVA (v8.0.2)<br>NovoCyte Quanteon (v1.4.1)
Data analysis	GraphPad Prism v. 9.0.0 , Adaptive ImmunoSEQ analyzer (v3), R version 3.6.1 and version 2.6.2, BD FACSDIVA v8.0.2, NovoExpress software v1.4.1. Immunospot software v5.1, R package "Matchlt", SAS version 9.4M5

For manuscripts utilizing custom algorithms or software that are central to the research but not yet described in published literature, software must be made available to editors and reviewers. We strongly encourage code deposition in a community repository (e.g. GitHub). See the Nature Research guidelines for submitting code & software for further information.

Data

Policy information about availability of data

All manuscripts must include a data availability statement. This statement should provide the following information, where applicable:

- Accession codes, unique identifiers, or web links for publicly available datasets
- A list of figures that have associated raw data
- A description of any restrictions on data availability

This clinical trial was registered on www.clinicaltrials.gov prior to patient enrollment (clinical trial identifer: NCT03047928)
Each vaccine was composed of 100 ug IO102, a 21-amino-acid peptide (DTLLKALLEIASCLEKALQVF) from the peptide IDO, and 100 ug IO103, a 19-amino-acid peptide (FMTYWHLLNAFTVTVPKDL) from the signal peptide of PD-L1.

The TCR sequencing data are available from Adaptive Biotechnologies. Upon request, the CCIT-DK office will provide a username and a password to access the designated data within approximately two to four weeks, https://www.herlevhospital.dk/ccit-denmark/find-us/Sider/Contact-information.aspx.
All requests for the remaining data including raw data and analyzed data and materials will within a reasonable time frame be reviewed by the CCIT-DK office https://www.herlevhospital.dk/ccit-denmark/find-us/Sider/Contact-information.aspx to verify whether the request is subject to any intellectual property or obligations. Patient-related data not included in the paper were generated as part of clinical trials and may be subject to patient confidentiality. Any data and materials that can be shared will be released via a Material Transfer Agreement.
The following databases were used in the study: https://research.regionh.dk/da/publications/the-danish-metastatic-melanoma-database-dammed(32749d99-095f-4cae-b5de-769bae27f01e).html

Field-specific reporting

Please select the one below that is the best fit for your research. If you are not sure, read the appropriate sections before making your selection.

☒ Life sciences ☐ Behavioural & social sciences ☐ Ecological, evolutionary & environmental sciences

For a reference copy of the document with all sections, see nature.com/documents/nr-reporting-summary-flat.pdf

Life sciences study design

All studies must disclose on these points even when the disclosure is negative.

Sample size

This study originally aimed to include 30 aPD1 treatment naive patients. An amendment with addition of two other cohorts (B and C) with 10 patients in each cohort was done to evaluate the immune responses and clinical efficacy in aPD1 resistant patients (de-novo resistance and required resistance). The amendment with cohort B and C was approved after having included 18 patients in cohort A. The trial is still including patients in cohort B and C. All thirty patients in cohort A have been included and considered for the primary endpoint as well as immunological and clinical response.
Since the trial is a first-in-human study, formal sample size calculation cannot be performed. The primary objective was to evaluate safety of the combination therapy and 30 patients were deemed sufficient to evaluate safety. The treatment was considered safe if less than 20% experience grade 3-4 AE's. Based on the expected inclusion of patients in this single-center study in a period of 2-3 years the study aimed to include 30 patients in total in cohort A.
The first 6 patients in cohort A were evaluated for safety and tolerability in phase I before the remaining 24 patients were included in phase II.

Data exclusions

Patients were considered evaluable for the primary endpoint if they recieved at least three series of therapy (both PD-L1/IDO peptide vaccine and nivolumab), which was specified prior enrollment in the protocol. 37 patients were screened for eligability in cohort A and 30 patients were included and treated. All thirty patients recieved allocated intervention.

Replication

All the data provided in the article is replicable. Elispot and flowcytometry analyses done at National Center for Cancer Immune Therapy (CCIT-DK) are described in detail in our methods section with references and we have documented the source of material used.
All ELISPOT assays were performed using a sufficient number of technical replicates using at least two different cell/well concentrations were made to allow for a clear confirmation, interpretation and statistical analysis of the data. In few minor cases, where result interpretation was complicated by the factors that could potentially influence the outcome of the readout, the assay has been replicated to confirm the result.
The majority of the intracellular staining (ICS) assay experiments shown in figures 3d, 4e,f, extended figure 9, 10 b,c,d has been successfully replicated at least twice. All flow cytometry analysis performed using proper assay controls to ensure clear interpretation of the results.
Due to limited patient material availability ICS data using patient PBMCs ex vivo could not be replicated.
T cell receptor sequencing (adaptive biotechnologies), RNA gene analysis (Nanostring), immunohistochemistry (Haliodx) were performed once due to clinical sample availability.

Randomization

This was a single-arm phase I/II study. No randomization was performed.

Blinding

All patients were allocated to recieve the same treatment (PD-L1/IDO peptide vaccine and nivolumab) and blinding was therefore not performed.

Reporting for specific materials, systems and methods

We require information from authors about some types of materials, experimental systems and methods used in many studies. Here, indicate whether each material, system or method listed is relevant to your study. If you are not sure if a list item applies to your research, read the appropriate section before selecting a response.

Materials & experimental systems

n/a	Involved in the study
☐	☒ Antibodies
☐	☒ Eukaryotic cell lines
☒	☐ Palaeontology and archaeology
☒	☐ Animals and other organisms
☐	☒ Human research participants
☐	☒ Clinical data
☒	☐ Dual use research of concern

Methods

n/a	Involved in the study
☒	☐ ChIP-seq
☐	☒ Flow cytometry
☒	☐ MRI-based neuroimaging

Antibodies

Antibodies used

Antibodies used for flowcytometry:
Mouse anti-human CD3 (APC-H7-conjugated) clone: SK7 dilution: 1:20 (cat. 560275), BD
Mouse anti-human CD3 (PE-CF594-conjugated) clone: UCHT1 dilution 0.8:30 (cat. 562280), BD
Mouse anti-human CD4 (PerCP-conjugated) clone: SK3 dilution: 1:5 (cat. 345770), BD
Mouse anti-human CD4 (BV711-conjugated) clone: SK3 dilution: 1:30 (cat. 563028), BD
Mouse anti-human CD8 (FITC-conjugated) clone: SK1 dilution: 1:5 (cat. 345772), BD
Mouse anti-human CD8 (Qdot605-conjugated) clone: 3B5 dilution: 1:150 (cat. Q10009), ThermoFischer
Mouse anti-human CD107a (PE-conjugated) clone: H4A3 dilution: (cat. 555801), BD
Mouse anti-human CD107a (BV421-conjugated) clone H4A3 dilution: 3:50 (cat. 328626), BD
Mouse anti-human IFN-γ (APC-conjugated) clone: 25723.11 (cat. 341117), BD
Mouse anti-human IFN-γ (PE-Cy7-conjugated) clone: B27 dilution: 1.5:20 (cat: 557643), BD
Mouse anti-human TNF (BV421-conjugated) clone: Mab11 dilution 1:100 (cat. 562783), BD
Mouse anti-human TNF (APC-conjugated) clone: Mab11 dilution 1:20 (cat. 554514), BD
Mouse anti-human CD137 (PE-conjugated) clone: 4B4-1 (RUO) dilution: 1:20 (cat. 555956), BD
Mouse anti-human PD-L1 (PE-Cy7-conjugated) clone: MIH1 (RUO) dilution: 1:23 (cat. 558017), BD
Mouse anti-human HLA II (FITC-conjugated) clone: Tu39 (RUO) dilution: 1:23 (cat. 555558), BD

Anti-HLA-DR antibody (unconjugated) clone: L243 dilution: 1:500 (cat: ab136320), Abcam
Anti-HLA-DQ antibody (unconjugated) clone: SPV-L3 dilution: 1:500 (cat. ab23632), Abcam
Anti-HLA-DP anitbody (unconjugated) clone: B7/21 dilution: 1:500 (cat. ab20897), Abcam

Antibodies used for IHC:
Anti-CD3 clone: HDX2 Provider: HalioDx ref: HD-FG-000013 lot: 10931065/10636667 concentration: 0,25µg/mL U
Anti-CD8 clone: HDX3 Provider: HalioDx ref: HD-FG-000019 lot: 10931069/10337710/10639301 concentration: 0,5µg/mL
IDO Monoclonal Antibody clone: VINC3IDO Provider: Thermofischer scientific ref: 14-9750-82 lot: E25003-101 conc: 0,05µg/mL
Anti-HLA Class 1 ABC antibody clone: EMR8-5 Provider: Abcam ref: ab70328 lot: GR3248333/GR3186494 concentration: 0.5 µg/mL
HLA-DR/DP/DQ/DX clone: CR3/43 Provider: Santa Cruz ref: sc-53302 lot: L1714 concentration: 1µg/mL
Anti-PD-L1 clone: HDX3 Provider: HalioDx ref: HD-FG-000035 lot: 106312810/106312816 concentration: 3,3µg/mL

Validation

Validation of antibodies used in flowcytometry:
All antibodies used in the flowcytometry experiments reported in the manuscript were purchased from commercial vendors and were validated by the vendors. We did not perform independent validation of these comercially available antibodies.
Mouse anti-human CD3 validated for flow cytometry on human thymocytes. Mouse anti-human CD4 validated for flowcytometry on human peripheral blood T cells, Mouse anti-human CD8 validated for flowcytometry on human peripheral blood T cells. Mouse anti-human CD107a validated for intracellular staining by flow cytometry in human adult adherent peripheral blood cells. Mouse anti-human IFN-γ validated for intracellular staining by flowcytometry in recombinant human IFN-γ. Mouse anti-human TNF validated for intracellular staining by flowcytomety in recombinant human TNF. Mouse anti-human CD137 validated for flow cytometry.

The antibody concentrations for staining were optimized by titrating down each reagent starting at the manufacturer`s recommendation. The optimal amounts of the reagents were defined by minimal unspecific shift of the negative population and a maximal separation of the negative and positive population.

IHC staining were performed at HalioDx Services GCP Laboratory. Specificity of each staining has been validated at HalioDx prior to be used and a medical pathologist reported a score.
A positive control slide is systematically used in each run.

Eukaryotic cell lines

Policy information about cell lines

Cell line source(s)

DSMZ-German Collection of Microorganisms and Cell Cultures GmbH

Authentication	cell line purchased from a commercial provider in 2019. Documentation for cell line authentication provided upon purchase
Mycoplasma contamination	proof of negative mycoplasma test provided by DSZM upon purchase of the cell line.
Commonly misidentified lines (See ICLAC register)	MonoMac6

Human research participants

Policy information about studies involving human research participants

Population characteristics	Eligable patients were ≥ 18 with locally advanced or stage IV melanoma according to AJCC, a least one measurable lesion according to RECIST 1.1 and Eastern Cooperative Oncology Group (ECOG). Main exclusion criteria were prior treatment with aPD-1 therapy (cohort A), CNS metastases >1 cm, severe comorbidities and active autoimmune disease.<br><br>Patients enrolled: 30<br>Patients treated with the IDO/PD-L1 vaccine and nivolumab: 30<br>Age (n=30): mean 70 years, range (46-85)<br>Male (n=30): 55%<br>ECOG- Performance status 0 (n=30): 87%<br>M-stage: (n=30) M1a: 20%, M1b:20% M1c:60%<br>PD-L1 >1% (n=30): 57%<br>Previsous systemic treatment (n=30): 10% have been treated with Ipilimumab, remaining patients are treatment naive (90%).
Recruitment	Patients in cohort A were newly referred to the oncology department at Herlev and Gentofte Hospital (Denmark) who were candidates for anti-PD1 monotherapy. Patients in cohort A were all recruited from our center by oncologist who daily treat patients with metastatic melanoma; the Oncology department at Herlev an Gentofte Hospital, Denmark.
Ethics oversight	The study was conducted according to the Declaration of Helsinki and Good Clinical Practice (GCP) and monitored by the GCP-unit, Copenhagen, Denmark. The protocol was approved by the Ethical Committee of the Capital region of Denmark (H-17000988), the Danish Medical Agencies (2017011073) and the Capital Region of Denmark Data Unit (P-2019-172). The study was registered at ClinicalTrials.gov, identifier: NCT03047928 and EudraCT no: 2016-0004527-23. All patients provided written informed consent.

Note that full information on the approval of the study protocol must also be provided in the manuscript.

Clinical data

Policy information about clinical studies

All manuscripts should comply with the ICMJE guidelines for publication of clinical research and a completed CONSORT checklist must be included with all submissions.

Clinical trial registration	ClinicalTrials.gov, identifier: NCT03047928
Study protocol	The study protocol will be uploaded with initial submission and made available upon request.
Data collection	Toxicity data was collected from the date of informed consent up to 6 months after the last dose of the IDO/PD-L1 peptide vaccine and nivolumab (cycle 24) during visits at the department of oncology, Herlev and Gentofte Hospital, Denmark. Clinical data was collected from the time of informed consent and up to 3.5 years after first dose in the eCRF (OpenClinica v1). Blood and tumour samples were collected in clinic visits at the department of oncology at Herlev and Gentofte Hospital and were stored at National Center for Cancer Immune Therapy (CCIT-DK).<br>The first patient was enrolled December 2017 and the last patient in cohort A was enrolled in June 2020. Data collection for the current analysis was performed on the the 5th of October 2020. eCRF database is located at CCIT-DK, Herlev University Hospital, Herlev, Denmark. Blood samples for immunologic analysis were collected at baseline, at cycle 3, 6, 12, 18 and 24 on vaccination and 3 and 6 months after vaccination. Tumour needle biopsies were collected at baseline and after 6th cycle when assessable. All data was collected at CCIT-DK, Herlev University Hospital, Denmark.
Outcomes	The primary endpoint was safety and tolerability which was monitored closely with changes in physical examination, clinical laboratory analyses and reported adverse events up to 6 months after the last dose of IDO/PD-L1 vaccine. Adverse events were assessed according to CTCAE v. 5.0. Safety were evaluated after the first three patients had recieved at least three series of treatment, and the ethics board approved further inclusion. Ethics board approval was repeated after 6 patients, and the final 24 patients were hereafter included.<br>Secondary outcomes included efficacy according to RECIST 1.1. Progression free survival and overall survival was also assessed. Immunologic analyses of vaccine specific responses detected by ELispot and intracellular cytokine staining by flowcytometry was assessed. Furthermore, immunological and genomic findings, including RNA gene signature, IHC of tumour biopsies to asses changes in the tumor microenvironemt and T-cell receptor (TCR) clonality.

Flow Cytometry

Plots

Confirm that:

☒ The axis labels state the marker and fluorochrome used (e.g. CD4-FITC).

☒ The axis scales are clearly visible. Include numbers along axes only for bottom left plot of group (a 'group' is an analysis of identical markers).

☒ All plots are contour plots with outliers or pseudocolor plots.

☒ A numerical value for number of cells or percentage (with statistics) is provided.

Methodology

Sample preparation	IDO and PD-L1 specific T cells were isolated from peptide stimulated in vitro PBMC cultures on day 14-15 after stimulation or in vitro expanded SKILs cultures using REP protocol with irradiated feeder cells and high dose IL-2 (6000Uml) for a minimum of 14 days before functional testing.<br><br>To asses ex vivo T cell reactiviy to IDO and PD-L1 peptides in patients PBMCs, cells were washed an rested for 1-2 days in media containing DNase followed by surface and intracellular staining.
Instrument	FACSCantoTM II (BD Biosciences) and NovoCyte Quanteon (ACEA Biosciences)
Software	BD FACSDiva software version 8.0.2. and NovoExpress software v.1.4.1
Cell population abundance	the purity of the samples was deterimined by staining for CD3 in combination with CD4/CD8. All sorted and expanded samples were >95% CD3+. Therefore to reflect the purity of the sorted and expanded specific T cell cultures, the percentage of peptide/vaccine specific T cells is represented as % of the total CD4+ or CD8+ T cells in a given culture
Gating strategy	Extended figure 10a<br>FSC-A x SCC-A for lymphocyte gate --><br>FSC-A x FSC-H for singlets gate --><br>SSC-A x dead marker -BV510 for live cells --><br>FSC-A x CD3 APC-H7 for CD3+ T cells --><br>CD8-FITC x CD4-PERCP for CD4+ and CD8+ T cells --><br>TNF-BV421 x IFNg APC quadrant gate seperating double positive, single positive and double negative cells.<br>and CD4/CD8 x CD107a-PE<br><br>Extended figure 10b.<br>FSC-A x SCC-A for lymphocyte gate --><br>FSC-A x FSC-H for singlets gate --><br>FSC-HA x dead marker - NIR - APC-Cy7 for live cells --><br>SSC-H-A x CD3 PE-CF594 for CD3+ T cells --><br>CD8-Qdotx CD4-BV711 for CD4+ and CD8+ T cells --><br>TNF-APC x IFNg PE-Cy7 quadrant gate seperating double positive, single positive and double negative cells.<br>and CD4/CD8 x CD107a-BV421 and CD4/CD8 x CD137-PE

☒ Tick this box to confirm that a figure exemplifying the gating strategy is provided in the Supplementary Information.
